# Global Natural Products Social (GNPS)-Based Molecular-Networking-Guided Isolation of Phenolic Compounds from *Ginkgo biloba* Fruits and the Identification of Estrogenic Phenolic Glycosides

**DOI:** 10.3390/plants12233970

**Published:** 2023-11-25

**Authors:** Chen Huo, Quynh Nhu Nguyen, Akida Alishir, Moon-Jin Ra, Sang-Mi Jung, Jeong-Nam Yu, Hui-Jeong Gwon, Ki Sung Kang, Ki Hyun Kim

**Affiliations:** 1 School of Pharmacy, Sungkyunkwan University, Suwon 16419, Republic of Korea; huochen_0213@163.com (C.H.); akida.alishir@gmail.com (A.A.); 2 College of Korean Medicine, Gachon University, Seongnam 13120, Republic of Korea; quynhnhunguyen.nnq@gmail.com; 3 Hongcheon Institute of Medicinal Herb, Hongcheon-gun 25142, Republic of Korea; ramj90@himh.re.kr (M.-J.R.); sgmo77@naver.com (S.-M.J.); 4 Nakdonggang National Institute of Biological Resources, Sangju 37242, Republic of Korea; susia000@nnibr.re.kr; 5 Advanced Radiation Technology Institute, Korea Atomic Energy Research Institute, Jeongeup 56212, Republic of Korea; hjgwon@kaeri.re.kr

**Keywords:** *Ginkgo biloba* fruits, GNPS-guided isolation, syringin, estrogen receptor, estrogenic activity

## Abstract

*Ginkgo biloba* L. stands as one of the oldest living tree species, exhibiting a diverse range of biological activities, including antioxidant, neuroprotective, anti-inflammatory, and cardiovascular activities. As part of our ongoing discovery of novel bioactive components from natural sources, we directed our focus toward the investigation of potential bioactive compounds from *G. biloba* fruit. The profiles of its chemical compounds were examined using a Global Natural Products Social (GNPS)-based molecular networking analysis. Guided by this, we successfully isolated and characterized 11 compounds from *G. biloba* fruit, including (*E*)-coniferin (**1**), syringin (**2**), 4-hydroxybenzoic acid 4-*O*-β-D-glucopyranoside (**3**), vanillic acid 4-*O*-β-D-glucopyranoside (**4**), syringic acid 4-*O*-β-D-glucopyranoside (**5**), (*E*)-ferulic acid 4-*O*-β-D-glucoside (**6**), (*E*)-sinapic acid 4-*O*-β-D-glucopyranoside (**7**), (1′*R*,2′*S*,5′*R*,8′*S*,2′*Z*,4′*E*)-dihydrophaseic acid 3′-*O*-β-D-glucopyranoside (**8**), eucomic acid (**9**), rutin (**10**), and laricitrin 3-rutinoside (**11**). The structural identification was validated through a comprehensive analysis involving nuclear magnetic resonance (NMR) spectroscopic data and LC/MS analyses. All isolated compounds were evaluated using an E-screen assay for their estrogen-like effects in MCF-7 cells. As a result, compounds **2**, **3**, **4**, **8**, and **9** promoted cell proliferation in MCF-7 cells, and these effects were mitigated by the ER antagonist, ICI 182,780. In particular, cell proliferation increased most significantly to 140.9 ± 6.5% after treatment with 100 µM of compound **2**. The mechanism underlying the estrogen-like effect of syringin (**2**) was evaluated using a Western blot analysis to determine the expression of estrogen receptor α (ERα). We found that syringin (**2**) induced an increase in the phosphorylation of ERα. Overall, these experimental results suggest that syringin (**2**) can potentially aid the control of estrogenic activity during menopause.

## 1. Introduction

Estrogen replacement therapy (ERT) is often considered an effective means of relief and prevention for women experiencing postmenopausal conditions [1,2]. However, prolonged use of ERT may lead to side effects, including breast cancer, heart disease, and stroke [3,4]. Consequently, there has been a recent emphasis on the search for compounds that mimic estrogenic effects. Phytoestrogens have emerged as promising candidates in this area due to their numerous pharmaceutical benefits for treating postmenopausal symptoms by acting as either agonists or antagonists on estrogen receptors [5]. Phytoestrogens, commonly referred to as “dietary estrogens”, are naturally occurring compounds derived from plants. These substances have the ability to bind to estrogen receptors, thereby generating estrogen-like effects that can effectively alleviate menopausal symptoms, such as hot flashes, night sweats, mood swings, depression, and nervous tension [6,7,8]. While various types of phytoestrogens, including flavonoids, lignans, and anthocyanins, have already been documented, there is an ongoing need to discover more potent and safer phytoestrogens for use in clinical settings [9,10,11].

*Ginkgo biloba* L., commonly known as the Ginkgo tree or maidenhair tree, is extensively cultivated in Asian countries [12,13,14]. As an ancient relic from the distant past, *G. biloba* stands as one of Earth’s oldest living tree species, earning its renowned status as “a living fossil” [15,16]. The medicinal use of *G. biloba* has ignited research interest, especially regarding its various parts, which are employed in orthodox or traditional medicine for treating diseases, attributed to the presence of numerous bioactive compounds [17,18]. Extensive investigations into *G. biloba* have unveiled a diverse array of phytochemicals within its composition. Notably, each component contributes to its pharmacological potential and therapeutic value, encompassing flavonoids, fatty acids, proanthocyanidins, terpenes, polysaccharides, and trilactones [19,20]. Furthermore, modern pharmacological studies have highlighted its beneficial effects, including anti-inflammatory, photoprotective, hepatoprotective, cardioprotective, antioxidant, anti-depressant, and anti-neurodegenerative properties [21,22,23,24]. It has been reported that *G. biloba* extract exhibits estrogenic activities, potentially linked to human breast cancer cells [25,26]. Consequently, our interest lies in discovering the active natural components responsible for these activities, guided by their biological effects and Global Natural Product Social (GNPS)-based molecular networking.

GNPS-based molecular networking has proven to be invaluable for the analysis of non-targeted mass spectrometry data across various fields [27,28,29]. It is recognized as an efficient tool for identifying and visualizing metabolites from natural extracts by matching experimental MS^2^ spectra against MS^2^ spectral libraries [30,31]. Recently, GNPS has emerged as a prominent tool for exploring and identifying active components in plants. In our recent investigations, we harnessed GNPS-guided isolation, leading to the discovery of four new madurastatin derivatives derived from the fungus *Actinomadura* sp. RB99 [32]. This outcome highlights the power of GNPS as an invaluable tool for discovering new natural compounds from natural sources. Additionally, in our recent study, we identified eight compounds from the mushroom *Armillariella tabescens* (Scop.) Sing. through bioactivity-guided fractionation. Notably, it was found that, among the identified compounds, (3*β*,5*α*,22*E*)-ergost-22-en-3-ol exhibited estrogenic activity as a mycoestrogen [33]. The results of these previous studies can serve as valuable knowledge and experience to inform future research endeavors.

As a part of our ongoing natural product discovery program [34,35,36,37,38] and our continued efforts to identify new bioactive compounds from *G. biloba* [39,40], we undertook a comprehensive investigation of the bioactive compounds present in *G. biloba* fruits using MS/MS-based GNPS approaches. The GNPS-based molecular networking approach guided the isolation of extracts from *G. biloba* fruits, ultimately resulting in the identification of 11 compounds (**1**–**11**). We characterized the structures of these isolated compounds using nuclear magnetic resonance (NMR) spectroscopy and data obtained from liquid chromatography (LC)–MS analysis. Subsequently, we assessed the estrogenic activity of these isolated compounds. In this report, we provide details on the isolation and structural characterization of these 11 compounds (**1**–**11**), as well as their estrogen-like effects in MCF-7 cells.

## 2. Results

### 2.1. GNPS-Molecular Networking Guided Isolation of Compounds

**Figure 1:**
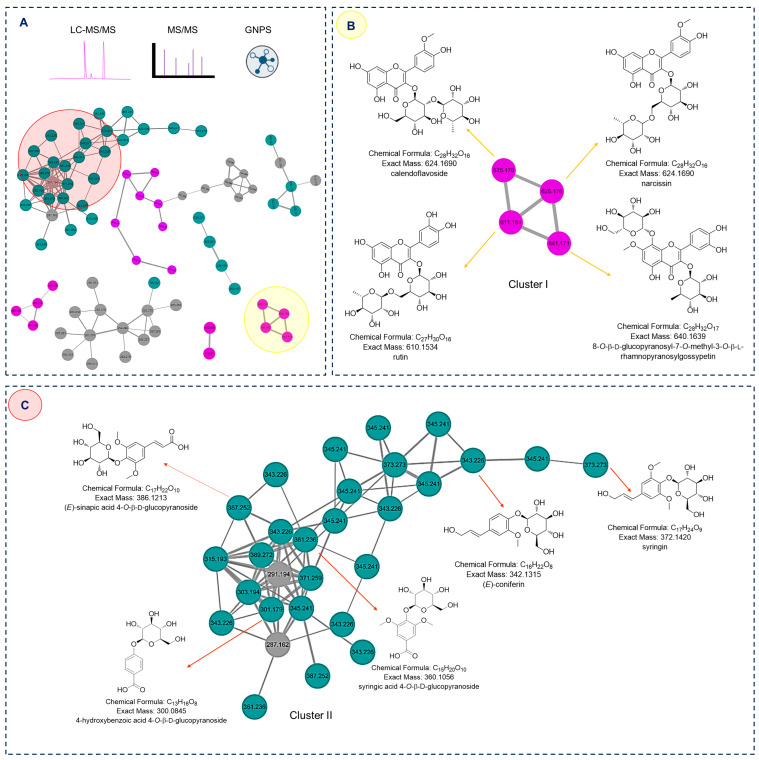
Molecular networking analysis and identification of compounds in the *n*-BuOH fraction derived from the MeOH extract of *G. biloba* fruits. (**A**) Molecular networking analysis of *n*-BuOH fraction and clusters of categorized compounds. (**B**) Zoomed-in molecular networking of flavonoid glycosides (Cluster I). (**C**) Zoomed-in molecular networking of phenolic glycosides and phenylpropanoid glycosides (Cluster II).

The LC-MS/MS data of *n*-BuOH fraction, which was the most abundant fraction derived from MeOH extract of *G. biloba* fruits, were subjected to analysis using the GNPS web platform (https://gnps.ucsd.edu, accessed on 21 November 2023). The result of the BuOH fraction was integrated by MolNetEnhancer workflow on the GNPS web platform, which automatically classified the chemical class of each cluster (Figure 1A). Among these clusters, Cluster I, represented by two precursor ions of *m/z* 625.176 [M+H]^+^, were unequivocally annotated as calendoflavoside and narcissin, respectively (Figure 1B). In addition, the precursor ions of *m*/*z* 611.161 and 641.171 were annotated as rutin and 8-*O*-β-D-glucopyranosyl-7-*O*-methyl-3-*O*-β-L-rhamnopyranosylgossypetin, respectively. Based on their structural characteristics, the phenolic glycosides and phenylpropanoid glycosides were categorized into Cluster II (Figure 1C). A precursor ion of *m*/*z* 343.226 was closely associated with (*E*)-coniferin, as revealed by GNPS analysis. Moreover, the presence of syringin detected from the analysis of the precursor ion at *m*/*z* 373.273, with a neutral additional 30 Da (a methoxy group), was consistently observed. The ion with *m*/*z* 301.179 was confidently identified as 4-hydroxybenzoic acid 4-*O*-β-D-glucopyranoside using the GNPS library. A precursor ion in Cluster II, with *m*/*z* 361.236, exhibited a significant increase of 60 Da (two methoxy groups) compared to *m*/*z* 301.179, implying the modification of 4-hydroxybenzoic acid 4-*O*-β-D-glucopyranoside in two methoxy groups, and this was identified as syringic acid 4-*O*-β-D-glucopyranoside. This clustering analysis offers strong evidence supporting the structural similarities and interconnected metabolic pathways among the compounds.

**Figure 2:**
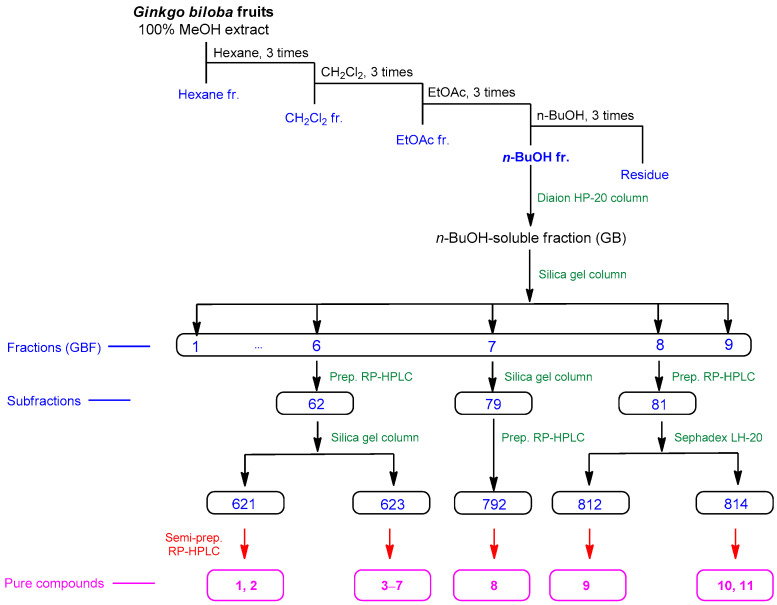
Separation of compounds **1**–**11** from MeOH extract of *G. biloba* fruits.

**Figure 3:**
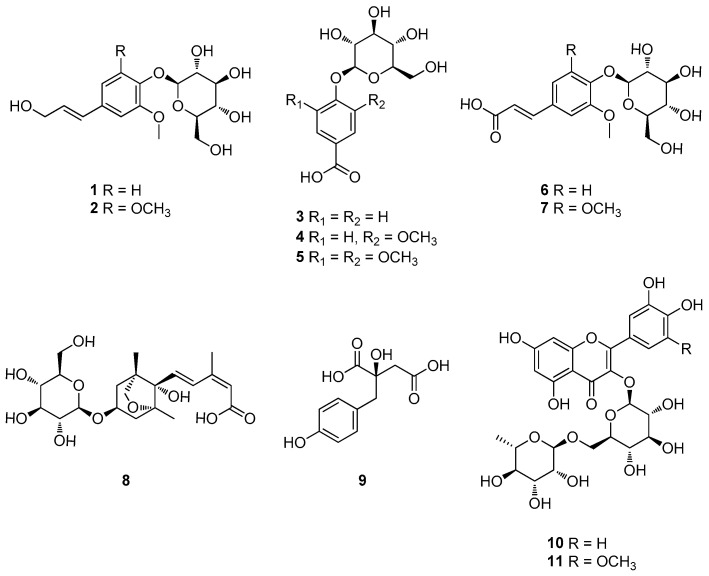
Chemical structures of compounds (**1**–**11**) isolated from *G. biloba* fruits.

As a result, it was revealed that the *n*-BuOH fraction contained flavonoid glycosides and phenolic glycoside with base peak ion MS observed in the Clusters I and II. Thus, the BuOH fraction was selected to focus on the isolation of flavonoid glycosides and phenolic glycosides (Figure 2). Additionally, the analysis of LC-MS using the house-built UV library database revealed that fractions GBF6––GBF8 were rich in flavonoid glycosides and phenolic glycosides. Consequently, seven phenolic glycosides, namely (*E*)-coniferin [(*E*) coniferyl alcohol glucoside] (**1**) [41], syringin [(*E)*-sinapyl alcohol 4-glucoside] (**2**) [42], 4-hydroxybenzoic acid 4-*O*-β-D-glucopyranoside (**3**) [43], vanillic acid 4-*O*-β-D-glucopyranoside (**4**) [44], syringic acid 4-*O*-β-D-glucopyranoside (**5**) [45], (*E*)-ferulic acid 4-*O*-β-D-glucoside (**6**) [46], and (*E*)-sinapic acid 4-*O*-β-D-glucopyranoside (**7**) [47]; one sesquiterpene glucoside, (1′*R*,2′*S*,5′*R*,8′*S*,2′*Z*,4′*E)*-dihydrophaseic acid 3′-*O*-β-D-glucopyranoside (**8**) [48]; a phenolic compound, eucomic acid (**9**) [49]; and two flavonoid derivatives, rutin (quercetin 3-*O*-rutinoside) (**10**) [50], and laricitrin 3-rutinoside (**11**) [51] were isolated from fractions GBF6–GBF8 (Figure 3 and Figure S1). Their structures were determined via a spectral analysis (Figures S2–S23), mainly ESI-MS, UV, and NMR experiments and comparison of their spectroscopic data with those previously reported. After obtaining the extract, it is beneficial to make predictions and selectively isolate the desired metabolites rather than conducting a separation without any prior information. GNPS-based molecular networking proves to be a valuable tool in this context. The compounds isolated in this study serve as notable examples of the efficacy of GNPS-molecular networking in guiding and enhancing the separation process.

### 2.2. Effects of Compounds ***1***–***11*** on the Proliferation of MCF-7 Cells

**Figure 4:**
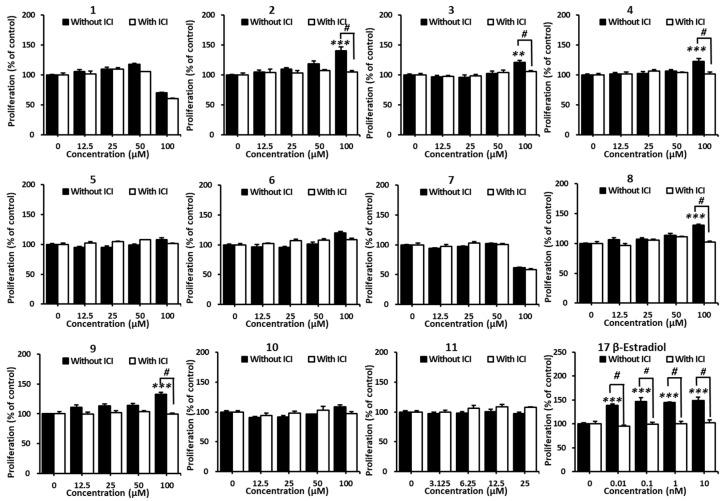
Effects of the isolated compounds **1**–**11** on MCF-7 cell viability. The proliferation of MCF-7 cells was assessed after 96 h of treatment. Cell viability is presented as mean ± standard error of the mean (SEM), ** *p* < 0.01 and *** *p* < 0.001 compared with the non-treated group. # *p* < 0.05 compared with the ICI-treated group at the same concentration of samples.

All the isolated compounds **1**–**11** were evaluated for their effects on MCF-7 cell proliferation to investigate estrogenic activity. Among the tested isolates, compounds **2**, **3**, **4**, **8**, and **9** increased cell proliferation in MCF-7 cells. Cell proliferation increased significantly to 140.9 ± 6.5%, 120.9 ± 3.7%, and 123.0 ± 4.2% after treatment with 100 µM of compounds **2**, **3**, and **4**, respectively. These effects were controlled by ICI 182,780, an ER antagonist (Figure 4). On the other hand, compounds **8** and **9** increased the cell growth of MCF-7 to 130.4 ± 1.8% and 132.6 ± 3.3% at 100 µM, respectively. However, cell growth remained unchanged when co-treated with ICI 182,780 (Figure 4). An active estrogen, 17β-estradiol, was used as a positive control to compare the effectiveness of the tested compounds. Starting from a concentration of 0.01 nM, 17β-estradiol significantly increased the cell viability of MCF-7 cells after 96 h of treatment, while maintaining it at the same level when co-cultured with ICI 182.780. These results demonstrate that these active compounds **2**, **3**, **4**, **8**, and **9** might be effective phytoestrogens, exhibiting E2-like activity in the proliferation of estrogen-receptor-positive breast cancer cells. Compound **2** showed a most significant effect compared to others; therefore, compound **2** was chosen for the further investigation of estrogenic-like effect mechanisms.

### 2.3. Effect of Compound ***2*** on the Protein Expression of Phospho-ERα and Erα

**Figure 5:**
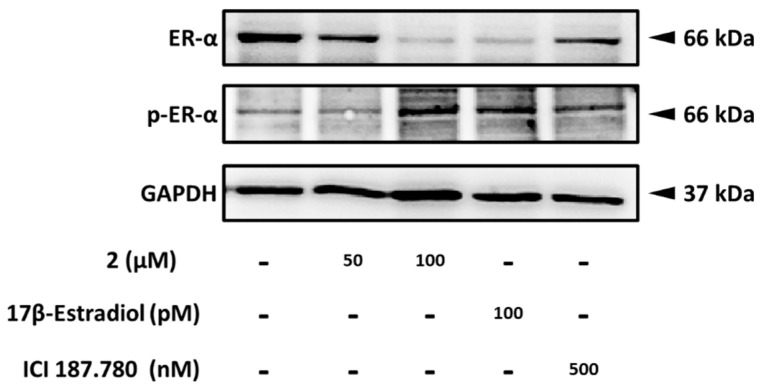
Effects of compound **2** on the regulation of estrogen receptor (ER)-α-dependent pathway. Western blotting of ER-α and phospho-ER-α (Ser118) proteins after 96 h treatment of MCF-7 cells with compound **2** and 17β-estradiol. Samples of 20 µg of protein were applied onto 10% SDS-PAGE.

To support the proliferation-promoting effect of compound **2**, we evaluated the expressions of ERα and p-ERα using a Western blot analysis. As a positive control, 17β-estradiol was used at 100 pM. Compared to untreated cells, treatment with 50 µM and 100 µM of compound **2** induced a concentration-dependent increase in the protein expression of p-ERα while reducing the expression of the regular form of ERα. The expression of the housekeeping gene GAPDH remained unchanged (Figure 5). As a positive control, 17β-estradiol at 100 pM had a similar effect as compound **2**, reducing ERα expression while inducing p-ERα protein expression and maintaining GAPDH at the same level as the non-treated group. Cells only treated with 500 nM of ICI 187,780 exhibited a reverse effect on the expressions of ERα and p-ERα, while GAPDH remained unchanged. These results provide insights into the estrogen-like effect mechanism of compound **2**, leading to the proliferation of estrogen receptor-positive human breast cancer MCF-7 cells.

## 3. Materials and Methods

### 3.1. Plant Material

In October 2019, *G. biloba* fruits were collected from the Sungkyunkwan University campus, situated in Suwon, Republic of Korea. The identification of the plant was confirmed by one of the authors (K.H.K.), and a voucher specimen (GBF-2019-10) was meticulously preserved in the herbarium of the School of Pharmacy at Sungkyunkwan University, Suwon, Korea.

### 3.2. Extraction and Isolation

A total of 4 kg of fresh *G. biloba* fruits were initially crushed and subjected to two successive extractions using 100% MeOH (8.0 L) over a 5-day period at room temperature. The resulting filtrate was then concentrated under reduced pressure via a rotary evaporator, yielding the MeOH extract (425.2 g). The crude MeOH extract was subsequently suspended in distilled water (700 mL) and partitioned with hexane, dichloromethane (CH_2_Cl_2_), ethyl acetate (EtOAc), and *n*-BuOH three times (700 mL for each partition). The organic phases were evaporated under vacuum conditions, not exceeding 40 °C, leading to the formation of four soluble fractions: hexane (8.2 g), CH_2_Cl_2_ (1.9 g), EtOAc (4.0 g), and *n*-BuOH-soluble fractions (28.8 g). A portion of the *n*-BuOH-soluble fraction (GB, 2.0 g) was subjected to a Diaion HP-20 column eluting H_2_O to remove the sugars. The resultant fraction was then subjected to chromatography on a silica gel column, with elution employing CH_2_Cl_2_/MeOH gradient steps, resulting in the collection of nine fractions (GBF1–GBF9) based on TLC analysis. Subsequently, the GBF6 fraction (366.4 mg) underwent preparative reversed-phase HPLC (prep. RP-HPLC), eluted using a gradient solvent system of MeOH/H_2_O (ranging from 40% to 100% MeOH) at a flow rate of 5 mL/min, leading to the separation of three subfractions (GBF61–GBF63). The GBF61 fraction (189.1 mg) was subjected to column chromatography on silica gel, employing CH_2_Cl_2_/MeOH gradient elution to produce six subfractions (GBF611–GBF616). The GBF611 fraction (88.3 mg) underwent further purification via semi-preparative reversed-phase HPLC (semi-prep. RP-HPLC) using a consistent solvent mixture of 27% MeOH/H_2_O at a flow rate of 2 mL/min, which resulted in the isolation of compounds **1** (2.3 mg) and **2** (1.4 mg). Similarly, the GBF613 fraction (46.0 mg) was subjected to semi-prep. RP-HPLC utilizing a 30% MeOH/H_2_O solvent system, leading to the isolation of compounds **3** (3.2 mg), **4** (2.2 mg), **5** (1.1 mg), **6** (0.8 mg), and **7** (1.0 mg). GBF7 fraction (257.0 mg) was subjected to column chromatography on silica gel, employing CH_2_Cl_2_/MeOH gradient steps, resulting in the collection of ten subfractions (GBF71–GBF79). The GBF79 fraction (99.7 mg) was further fractionated through prep. RP-HPLC, using a gradient solvent system of MeOH/H_2_O (ranging from 30% to 80% MeOH) at a flow rate of 5 mL/min, which led to the production of four subfractions (GBF791–GBF794). Compound **8** (2.3 mg) was successfully isolated from the GBF792 fraction utilizing the same semi-prep. RP-HPLC conditions with 18% MeOH/H_2_O solvent system. The GBF8 fraction (258.7 mg) underwent prep. RP-HPLC with a gradient solvent system of MeOH/H_2_O (ranging from 30% to 80% MeOH) at a flow rate of 5 mL/min, resulting in the production of four fractions (GBF81–GBF84). Among these fractions, GBF81 fraction was further purified through column chromatography on Sephadex LH-20 using 100% MeOH, generating four subfractions (GBF811–GBF814). The GBF812 fraction (67.1 mg) was subsequently subjected to semi-prep. RP-HPLC, employing a 35% MeOH/H_2_O solvent system, ultimately yielding compound **9** (11.3 mg). Finally, compounds **10** (1.3 mg) and **11** (0.9 mg) were isolated from GBF814 fraction (43.6 mg) using semi-prep. RP-HPLC with 35% MeOH/H_2_O solvent system.

### 3.3. LC-MS/MS Analysis

The *n*-BuOH fraction was dissolved in MeCN/H_2_O (9:1) at a concentration of 1.0 mg/mL and analyzed using an Agilent G6545B quadrupole time-of-flight (Q-TOF) mass spectrometer (Agilent Technologies, Santa Clara, CA, USA) equipped with a heated electrospray ion source (HESI). Chromatographic separation was carried out on an Acquity^®^ UPLC BEH reverse-phase C18 column (150 mm × 2.1 mm, 1.7 μM). The elution was achieved with a gradient of 0.1% formic acid in H_2_O (A) and MeCN (B), starting with 5% B and increasing to 100% B over 20 min, followed by a 3 min wash with 100% B and a 3 min re-conditioning step with 5% B, all at a flow rate of 0.3 mL/min. MS/MS spectra were acquired using electrospray ionization (ESI) in positive ion mode. A low collision energy of 6 eV was used for detecting precursor ions, and a high collision energy range of 20–40 eV was employed for fragmentation [52]. The RAW files generated were then converted into the open-source ‘.mzXML’ file format using the ProteoWizard MSConvert Version 3 Software. To facilitate file transfer, the recommended FTP client, WinSCP, was utilized to upload the files onto the GNPS platform. The acquired MS^2^ data underwent visualization using GNPS-based visualization tools. Subsequently, the molecular networks generated were exported from GNPS in ‘.graphml’ format and imported into Cytoscape for customized visualization and analysis.

### 3.4. Cell Culture

The ER-positive MCF-7 human breast epithelial cell line, sourced from the American Type Culture Collection (Manassas, VA, USA), was cultivated in Roswell Park Memorial Institute-1640 (RPMI1640) medium (Cellgro, Manassas, VA, USA). The culture medium was supplemented with 100 μg/mL streptomycin, 100 U/mL penicillin, and 10% fetal bovine serum (Gibco BRL, Grand Island, NY, USA). The MCF-7 cells were maintained in an incubator at 37 °C with a CO_2_ concentration of approximately 5%.

### 3.5. E-Screen Assay

MCF-7 cells were inoculated into 96-well plates at the density of 7.5 × 10^3^ cells in 100 μL per well with 95% relative humidity, 5% CO_2_, and 37 °C. After a 24 h incubation, the seeding medium was removed and replaced by the sample treatment with the concentrations of 12.5, 25, 50, and 100 μM in phenol red-free RPMI, which was supplemented with 10% charcoal-stripped heat-inactivated human serum (Innovative Research, Novi, MI, USA), 100 units/mL of penicillin, and 100 μg/mL of streptomycin (PS, Gibco BRL, Carlsbad, MD, USA). The treatment regimen was sustained for a duration of 96 h, and the assessment of cell proliferation was conducted using 10% of the Ez-Cytox cell proliferation assay kit (Daeil Lab Service Co., Seoul, Republic of Korea). The cells were incubated in the culture medium with the assay kit for 1 h, and subsequent measurements were obtained utilizing a microplate reader (PowerWave XS, Bio-Tek Instruments, Winooski, VT, USA).

### 3.6. Western Blot Analysis

MCF-7 cells were seeded into 60 mm culture plates at a density of 2 × 10^5^ cells. After a 24 h incubation, the cells were treated with samples in the treatment medium as previously described. The fresh treatment medium content samples were replaced after 48 h, and the treatment was continued for 96 h. Then, the treatment medium was discarded, and the cells were washed with Dulbecco’s phosphate-buffered saline (DPBS, Welgene Inc., Daegu, Republic of Korea). Subsequently, cells were harvested and lysed in 1× RIPA buffer (Tech & Innovation, Gangwon, Republic of Korea) supplemented with a proteinase inhibitor cocktail (Roche Diagnostics, Basel, Switzerland) to obtain whole-cell extracts. The protein content in each sample’s cell extract was quantified using the Pierce BCA protein assay kit (Thermo Fisher Scientific, Waltham, MA, USA). Equal amounts of proteins (20 µg/lane) were then separated using 10% sodium dodecyl sulfate-polyacrylamide gel electrophoresis and subsequently transferred onto polyvinylidene difluoride membranes. Next, the membranes were blocked with 5% skim milk in Tris-buffered saline (1×) for 1 h, and the separated proteins were identified via incubation with epitope-specific primary antibodies of the estrogen receptor (ERα), phospho-estrogen receptor (p-ERα), and GAPDH. Then, secondary antibodies were identified to recognize these primary antibodies (Cell Signaling Technology, Danvers, MA, USA). The membranes were visualized using a SuperSignal West Femto Maximum Sensitivity Substrate (Thermo Fisher Scientific) and captured using a FUSION Solo Chemiluminescence System (Vilber Lourmat Deutschland GmbH, Eberhardzell, Germany).

### 3.7. Statistical Analysis

All experiments were conducted in triplicate and repeated at least three times for robustness and reliability. The results are expressed as the mean ± standard error of the mean (SEM). Statistical significance was determined using a one-way analysis of variance (ANOVA) with subsequent multiple comparisons employing the Tukey post hoc test. A *p*-value of less than 0.05 was considered statistically significant. All statistical analyses were carried out using Prism version 8.1 (GraphPad Software, San Diego, CA, USA).

## 4. Discussion

Leaves of *G. biloba* have been used in traditional Chinese medicine for 5000 years [12]. Several secondary metabolites, including terpenoids, polyphenols, allyl phenols, organic acids, carbohydrates, fatty acids and lipids, inorganic salts, and amino acids, have been isolated from the *G. biloba* [40]. However, the main bioactive constituents are known as terpene trilactones and flavonoid glycosides, which are considered responsible for pharmacological activities, such as scavenging free radicals, lowering oxidative stress, reducing neural damage, reducing platelet aggregation, anti-inflammation, anti-tumor activities, and anti-aging effects [21,23,53,54,55,56]. The components and bioactive compounds of the leaves [57,58,59] and seeds [60] have been studied by many natural product chemists. *G. biloba* fruit is a rich source of nutrients and bioactive compounds [61]; however, *G. biloba* fruit metabolites have not been extensively explored. In this study, we investigated and isolated the potential bioactive compounds from the fruit extract using a Global Natural Products Social (GNPS)-based molecular networking analysis. Structural identification was validated through a comprehensive analysis involving mainly NMR spectroscopic data and LC/MS analyses. A range of compounds from *G. biloba* fruit were characterized, including (*E*)-coniferin (**1**), syringin (**2**), 4-hydroxybenzoic acid 4-*O*-β-D-glucopyranoside (**3**), vanillic acid 4-*O*-β-D-glucopyranoside (**4**), syringic acid 4-*O*-β-D-glucopyranoside (**5**), (*E*)-ferulic acid 4-*O*-β-D-glucoside (**6**), (*E*)-sinapic acid 4-*O*-β-D-glucopyranoside (**7**), (1′*R*,2′*S*,5′*R*,8′*S*,2′*Z*,4′*E*)-dihydrophaseic acid 3′-*O*-β-D-glucopyranoside (**8**), eucomic acid (**9**), rutin (**10**), and laricitrin 3-rutinoside (**11**). These compounds were subjected to estrogen-receptor-α-dependent signaling pathways in MCF-7 cells to evaluate their estrogenic activity. Among them, compounds **2**, **3**, **4**, **8**, and **9** showed potential estrogenic-like effects in MCF-7 human ER-positive breast cancer cells. Particularly, syringin (**2**) was the most promising among them at the same concentration compared to the other active compounds.

Phytoestrogens are naturally occurring compounds found in plants and classified into seven groups: isoflavones, flavones, flavanones, chalcones, coumestanes, lignanes, and stilbenes [62]. Natural compounds, such as phytoestrogens, have gained significant research interest due to their estrogenic activity and their biological relevance to women’s health [63]. Phytoestrogens have been considered in hormone replacement therapy because they mimic or modulate the action of endogenous estrogens, potentially preventing breast cancer development [63,64]. Moreover, the presence of a phenolic ring in these compounds allows them to bind to the estrogen receptor (ER), imitating the effects of estrogen [65]. In this study, syringin (**2**) promoted proliferation in human breast cancer MCF-7 cells by regulating the estrogen receptor pathway, specifically ER-α and p-ERα expression, a mechanism similar to that of estrogen, 17β-estradiol. Therefore, our study suggests that syringin (**2**) could be a potential phytoestrogen for estrogen replacement therapy in women experiencing menopausal symptoms.

Syringin, a phenylpropanoid glycoside found in various plant species, has garnered significant attention from researchers due to its potential health benefits, and its pharmacological properties have been extensively investigated. In 2015, one group summarized the activities of syringin, which include scavenging free radicals, protecting against neuronal cell damage, inhibiting apoptosis, exerting anti-diabetic effects, displaying anti-inflammatory potential, and acting as an anti-nociceptive and anti-allergic agent [66]. Additionally, a recent study revealed that syringin exerts anti-breast cancer effects through the PI3K-AKT and EGFR-RAS-RAF pathways in MDA-MB-231 and MCF-7 cells in in a culture medium with a high sugar and serum content supplemented with 12% fetal bovine serum (FBS) [67]. However, in the present study, we found that syringin promotes proliferation in human breast cancer MCF-7 cells by regulating the estrogen receptor pathway in charcoal-stripped fetal bovine serum. Considering the diverse pharmacological properties of syringin, it could be explored in the development of multi-target drugs. Its wide range of effects, from antioxidant and anti-inflammatory actions to its impact on cancer pathways, underscores its importance in the field of medicine and highlights the need for continued investigations to fully harness its therapeutic potential.

## 5. Conclusions

In conclusion, *G. biloba*, an ancient and resilient tree species, continues to reveal its pharmacological potential through a diverse array of biological activities. Our study, driven by the pursuit of novel bioactive compounds from natural sources, delved into *G. biloba* fruit, where the Global Natural Products Social (GNPS)-based molecular networking approach guided isolation led to the identification of 11 compounds (**1**–**11**). Moreover, these compounds were assessed for estrogenic activity via estrogen receptor α-dependent signaling pathways in MCF-7 cells. Remarkably, syringin (**2**) emerged as a standout, exhibiting robust estrogenic activity. The experimental results of this study suggest that syringin (**2**) has the potential to aid in the control of estrogenic activity during menopause.

## Data Availability

Data are available on request.

## Supplementary Materials

The following materials are available online at https://www.mdpi.com/article/10.3390/plants12233970/s1, Figure S1: Semi-preparative HPLC chromatogram of isolated compounds **1**–**11**; Figure S2: ^1^H-NMR (CD_3_OD, 850 MHz) spectrum of (*E*)-coniferin (**1**); Figure S3: UV chromatogram of LC/MS (A: 254 nm) and UV (B) and MS data (C: positive; D: negative) for **1**; Figure S4: ^1^H-NMR (CD_3_OD, 850 MHz) spectrum of syringin (**2**); Figure S5: UV chromatogram of LC/MS (A: 254 nm) and UV (B) and MS data (C: positive; D: negative) for **2**; Figure S6: ^1^H-NMR (CD_3_OD, 850 MHz) spectrum of 4-hydroxybenzoic acid 4-*O*-β-D-glucopyranoside (**3**); Figure S7: UV chromatogram of LC/MS (A: 254 nm) and UV (B) and MS data (C: positive; D: negative) for **3**; Figure S8: ^1^H-NMR (CD_3_OD, 850 MHz) spectrum of vanillic acid 4-*O*-β-D-glucopyranoside (**4**); Figure S9: UV chromatogram of LC/MS (A: 315 nm) and UV (B) and MS data (C: positive; D: negative) for **4**; Figure S10: ^1^H-NMR (CD_3_OD, 850 MHz) spectrum of syringic acid 4-*O*-β-D-glucopyranoside (**5**); Figure S11: UV chromatogram of LC/MS (A: 315 nm) and UV (B) and MS data (C: positive; D: negative) for **5**; Figure S12: ^1^H-NMR (CD_3_OD, 850 MHz) spectrum of (*E*)-ferulic acid 4-*O*-β-D-glucoside (**6**); Figure S13: UV chromatogram of LC/MS (A: 315 nm) and UV (B) and MS data (C: positive; D: negative) for **6**; Figure S14: ^1^H-NMR (CD_3_OD, 850 MHz) spectrum of (*E*)-sinapic acid 4-*O*-β-D-glucopyranoside (**7**); Figure S15: UV chromatogram of LC/MS (A: 315 nm) and UV (B) and MS data (C: positive; D: negative) for **7**; Figure S16: ^1^H-NMR (CD_3_OD, 850 MHz) spectrum of (1′*R*,2′*S*,5′*R*,8′*S*,2′*Z*,4′*E*)-dihydrophaseic acid 3′-*O*-β-D-glucopyranoside (**8**); Figure S17: UV chromatogram of LC/MS (A: 254 nm) and UV (B) and MS data (C: positive; D: negative) for **8**; Figure S18: ^1^H-NMR (CD_3_OD, 850 MHz) spectrum of eucomic acid (**9**); Figure S19: UV chromatogram of LC/MS (A: 254 nm) and UV (B) and MS data (C: positive; D: negative) for **9**; Figure S20: ^1^H-NMR (CD_3_OD, 850 MHz) spectrum of rutin (**10**); Figure S21: UV chromatogram of LC/MS (A: 254 nm) and UV (B) and MS data (C: positive; D: negative) for **10**; Figure S22: ^1^H-NMR (CD_3_OD, 850 MHz) spectrum of laricitrin 3-rutinoside (**11**); Figure S23: UV chromatogram of LC/MS (A: 254 nm) and UV (B) and MS data (C: positive; D: negative) for **11**; Figure S24: The uncropped Western blot gels; General Experimental Procedure.
