# Supplementary material for: Global Natural Products Social (GNPS)-Based Molecular-Networking-Guided Isolation of Phenolic Compounds from Ginkgo biloba Fruits and the Identification of Estrogenic Phenolic Glycosides
Source: Plants (Basel). 2023 Nov 25;12(23):3970. doi: 10.3390/plants12233970 (PMC10708316; doi:10.3390/plants12233970)
Supplement: Supplementary file 1 [file plants-12-03970-s001.zip › plants-2679755-supplementary.pdf]

Supplementary Material

Global Natural Products Social (GNPS)-Based Molecular-Networking-Guided Isolation of Phenolic Compounds from *Ginkgo biloba* Fruits and the Identification of Estrogenic Phenolic Glycosides

**Chen Huo [1,†], Quynh Nhu Nguyena [2,†], Akida Alishir [1], Moon-Jin Ra [3], Sang-Mi Jung [3], Jeong-Nam Yu [4],
Hui-Jeong Gwon [5], Ki-Sung Kang [2,*] and Ki-Hyun Kim [1,*]**

[1] School of Pharmacy, Sungkyunkwan University, Suwon 16419, Republic of Korea; huochen_0213@163.com (C.H.); akida.alishir@gmail.com (A.A.)
[2] College of Korean Medicine, Gachon University, Seongnam 13120, Republic of Korea; quynhnhunguyen.nnq@gmail.com
[3] Hongcheon Institute of Medicinal Herb, Hongcheon-gun 25142, Republic of Korea; ramj90@himh.re.kr (M.-J.R.); sgmo77@naver.com (S.-M.J.)
[4] Nakdonggang National Institute of Biological Resources, Sangju 37242, Republic of Korea; susia000@nnibr.re.kr
[5] Advanced Radiation Technology Institute, Korea Atomic Energy Research Institute, Jeongeup 56212, Republic of Korea; hjgwon@kaeri.re.kr
* Correspondence: kkang@gachon.ac.kr (K.-S.K.); khkim83@skku.edu (K.-H.K.); Tel.: +82-31-750-5402 (K.-S.K.); +82-31-290-7700 (K.-H.K.)
† These authors contributed equally to this work.

Figure S1. Semi-preparative HPLC chromatogram of isolated compounds **1**−**11**.. 4

Figure S2. ^{1}H-NMR (CD_3OD, 850 MHz) spectrum of (*E*)-coniferin (**1**). 5

Figure S3. UV chromatogram of LC/MS (A: 254 nm) and UV (B) and MS data (C: positive; D: negative) for **1**. 6

Figure S4. ^{1}H-NMR (CD_3OD, 850 MHz) spectrum of syringin (**2**). 7

Figure S5. UV chromatogram of LC/MS (A: 254 nm) and UV (B) and MS data (C: positive; D: negative) for **2**. 8

Figure S6. ^{1}H-NMR (CD_3OD, 850 MHz) spectrum of 4-hydroxybenzoic acid 4-*O*-β-D-glucopyranoside (**3**). 9

Figure S7. UV chromatogram of LC/MS (A: 254 nm) and UV (B) and MS data (C: positive; D: negative) for **3**. 10

Figure S8. ^{1}H-NMR (CD_3OD, 850 MHz) spectrum of vanillic acid 4-*O*-β-D-glucopyranoside (**4**). 11

Figure S9. UV chromatogram of LC/MS (A: 315 nm) and UV (B) and MS data (C: positive; D: negative) for **4**. 12

Figure S10. ^{1}H-NMR (CD_3OD, 850 MHz) spectrum of syringic acid 4-*O*-β-D-glucopyranoside (**5**). 13

Figure S11. UV chromatogram of LC/MS (A: 315 nm) and UV (B) and MS data (C: positive; D: negative) for **5**. 14

Figure S12. ^{1}H-NMR (CD_3OD, 850 MHz) spectrum of (*E*)-ferulic acid 4-*O*-β-D-glucoside (**6**). 15

Figure S13. UV chromatogram of LC/MS (A: 315 nm) and UV (B) and MS data (C: positive; D: negative) for **6**. 16

Figure S14. ^{1}H-NMR (CD_3OD, 850 MHz) spectrum of (*E*)-sinapic acid 4-*O*-β-D-glucopyranoside (**7**). 17

Figure S15. UV chromatogram of LC/MS (A: 315 nm) and UV (B) and MS data (C: positive; D: negative) for **7**. 18

Figure S16. ^{1}H-NMR (CD_3OD, 850 MHz) spectrum of (1'*R*,2'*S*,5'*R*,8'*S*,2'*Z*,4'*E*)-dihydrophaseic acid 3'-*O*-β-D-glucopyranoside (**8**). 19

Figure S17. UV chromatogram of LC/MS (A: 254 nm) and UV (B) and MS data (C: positive; D: negative) for **8**. 20

Figure S18. ^{1}H-NMR (CD_3OD, 850 MHz) spectrum of eucomic acid (**9**). 21

Figure S19. UV chromatogram of LC/MS (A: 254 nm) and UV (B) and MS data (C: positive; D: negative) for **9**. 22

Figure S20. ^{1}H-NMR (CD_3OD, 850 MHz) spectrum of rutin (**10**)...................................... 23

Figure S21. UV chromatogram of LC/MS (A: 254 nm) and UV (B) and MS data (C: positive; D: negative) for **10**. 24

Figure S22. ^{1}H-NMR (CD_3OD, 850 MHz) spectrum of laricitrin 3-rutinoside (**11**). 25

Figure S23. UV chromatogram of LC/MS (A: 254 nm) and UV (B) and MS data (C: positive; D: negative) for **11**. 26

Figure S24. The uncropped western blot gels ……………………..………………..……...27

General Experimental Procedure ……………………………………...………………..….28

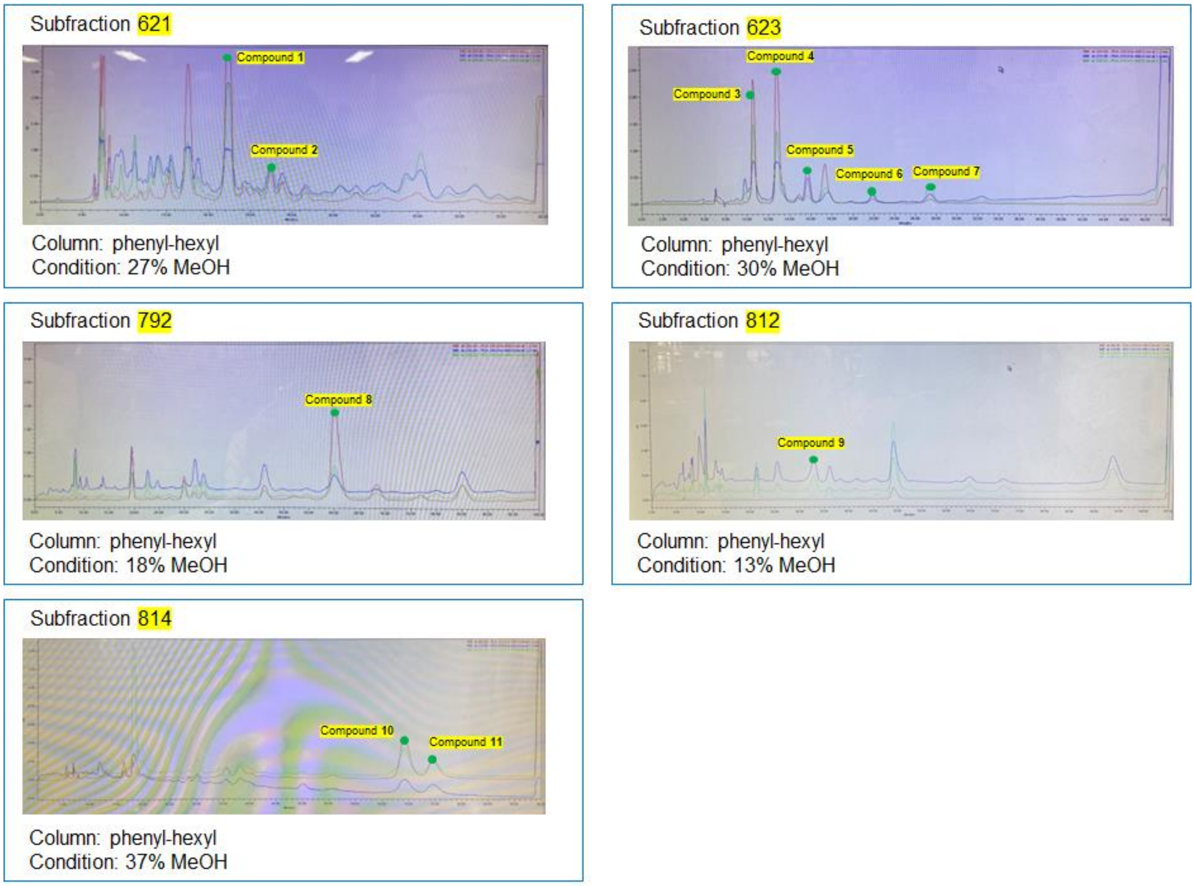

Figure S1. Semi-preparative HPLC chromatogram of isolated compounds **1**−**11**.

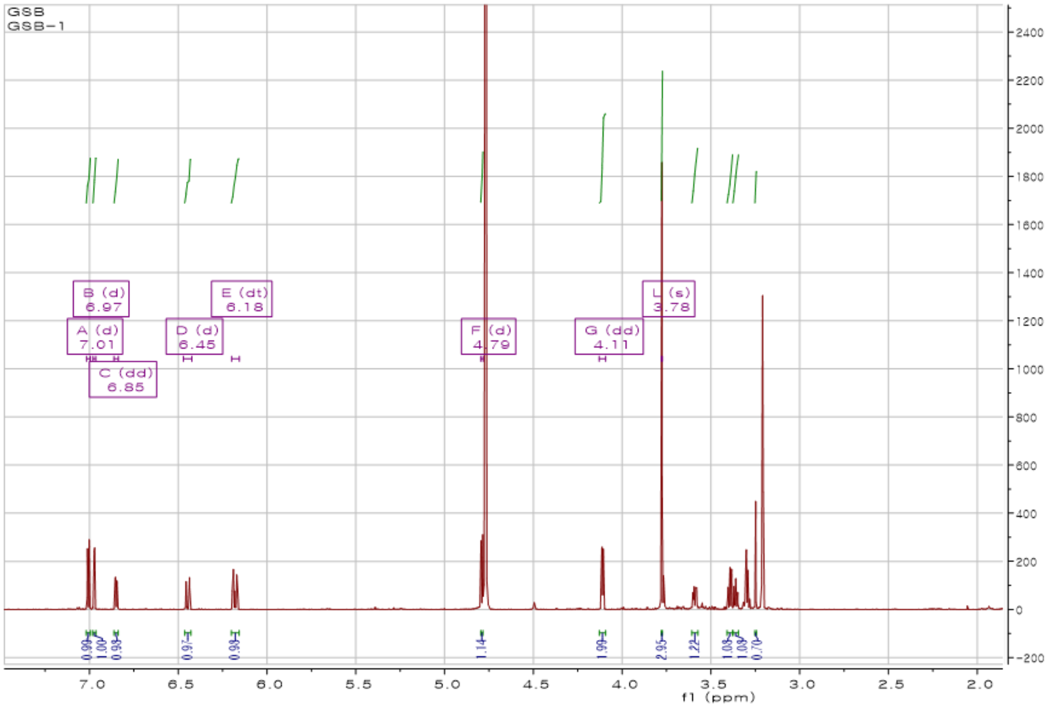

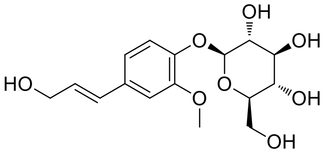

1

Figure S2. ^{1}H-NMR (CD_3OD, 850 MHz) spectrum of (*E*)-coniferin (**1**).

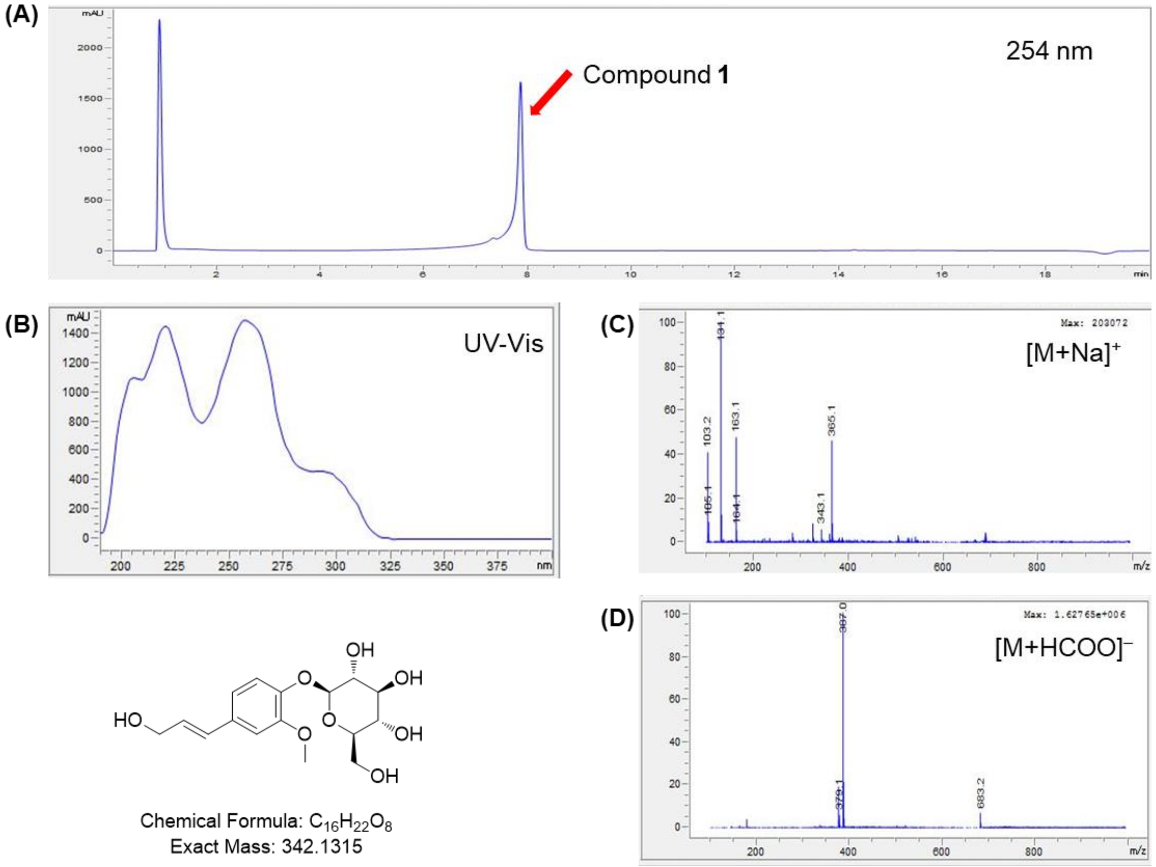

Figure S3. UV chromatogram of LC/MS (A: 254 nm) and UV (B) and MS data (C: positive; D: negative) for **1**.

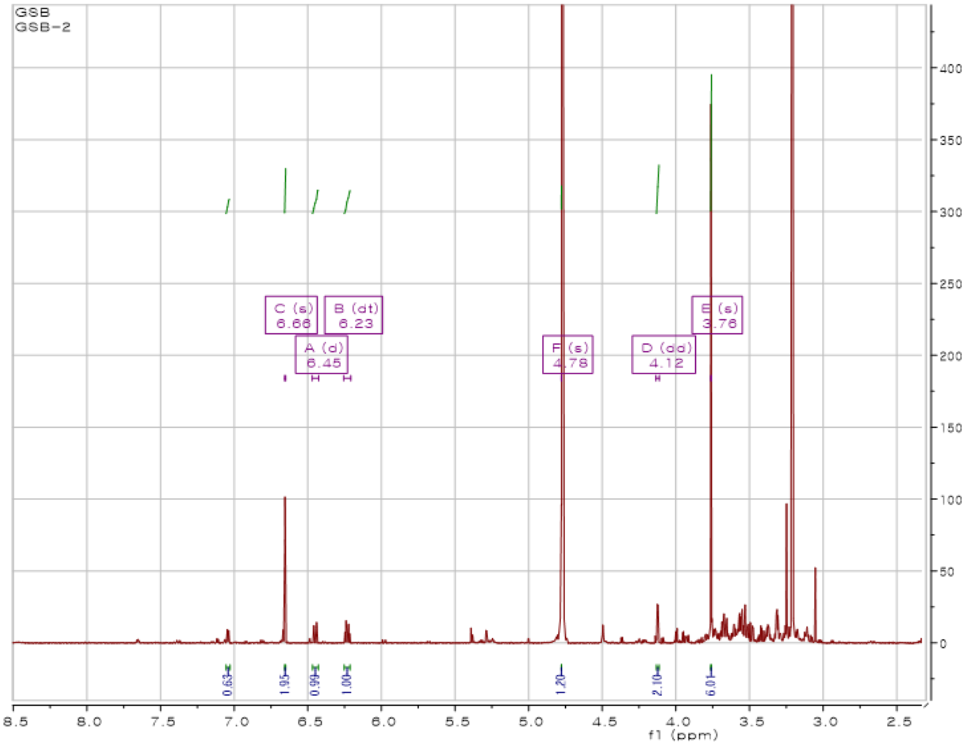

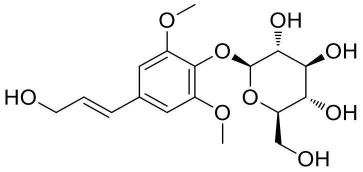

2

Figure S4. ^{1}H-NMR (CD_3OD, 850 MHz) spectrum of syringin (**2**).

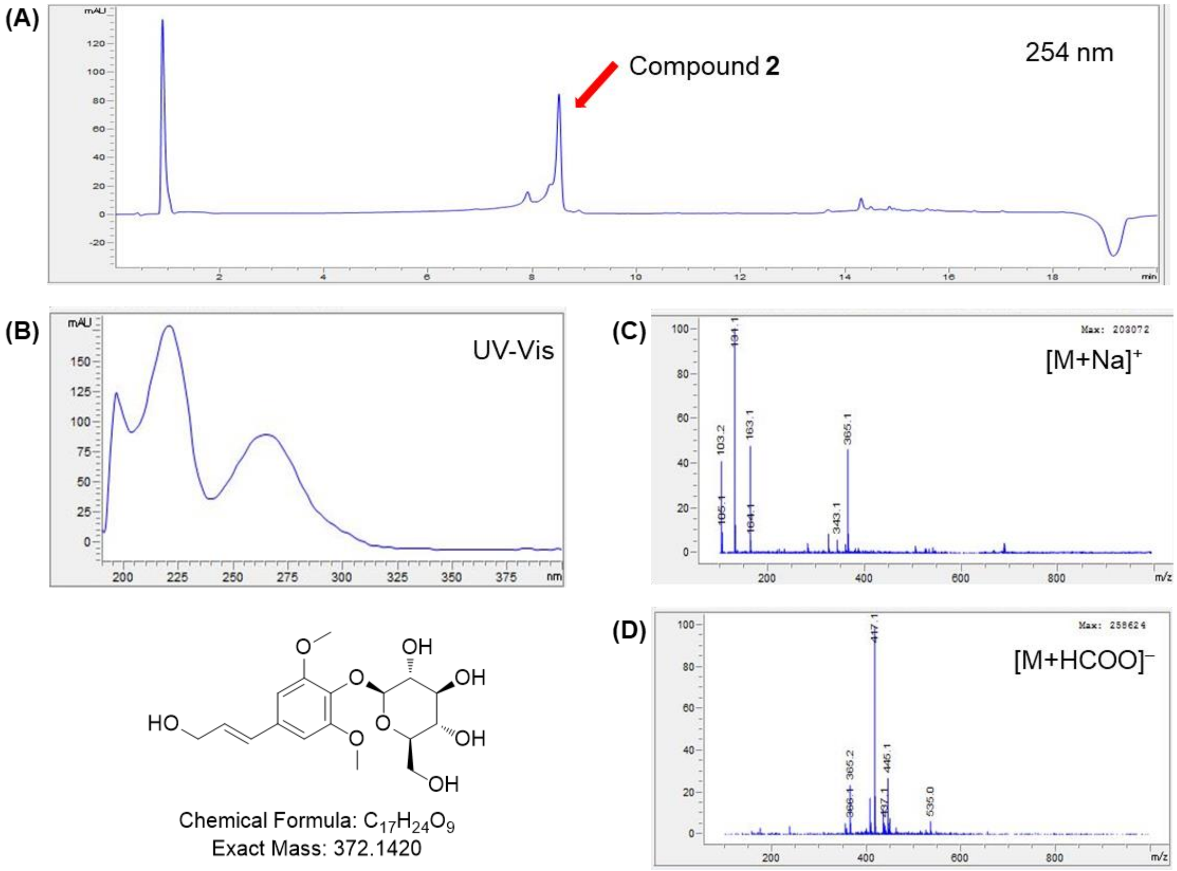

Figure S5. UV chromatogram of LC/MS (A: 254 nm) and UV (B) and MS data (C: positive; D: negative) for **2**.

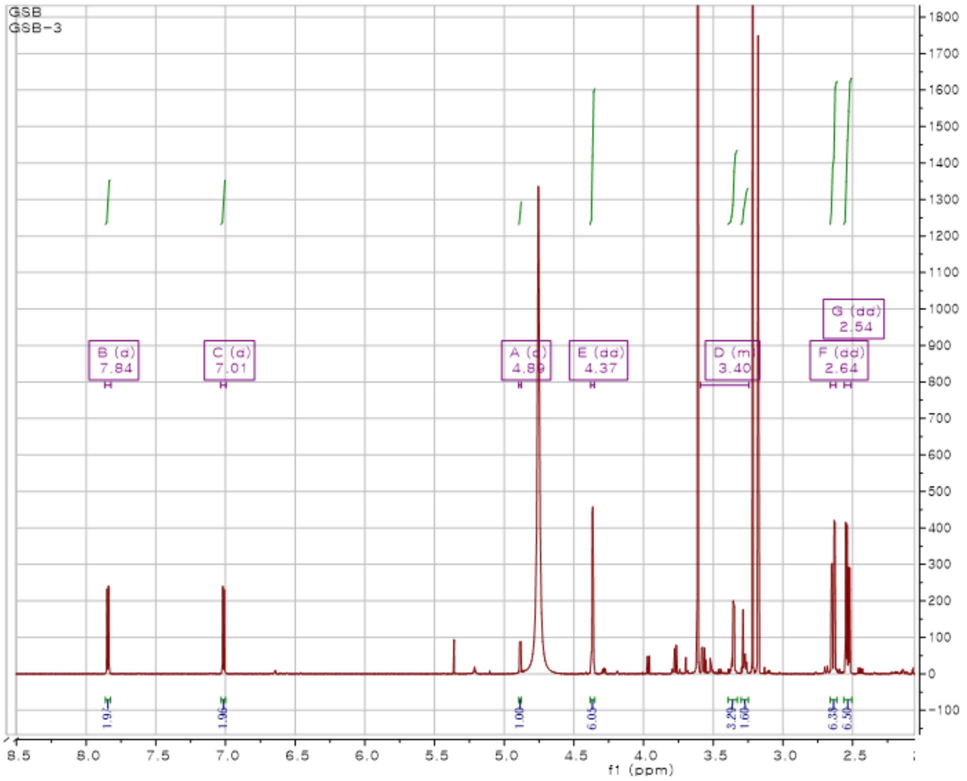

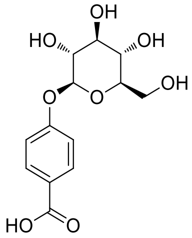

3

Figure S6. ^{1}H-NMR (CD_3OD, 850 MHz) spectrum of 4-hydroxybenzoic acid 4-*O*-β-D-glucopyranoside (**3**).

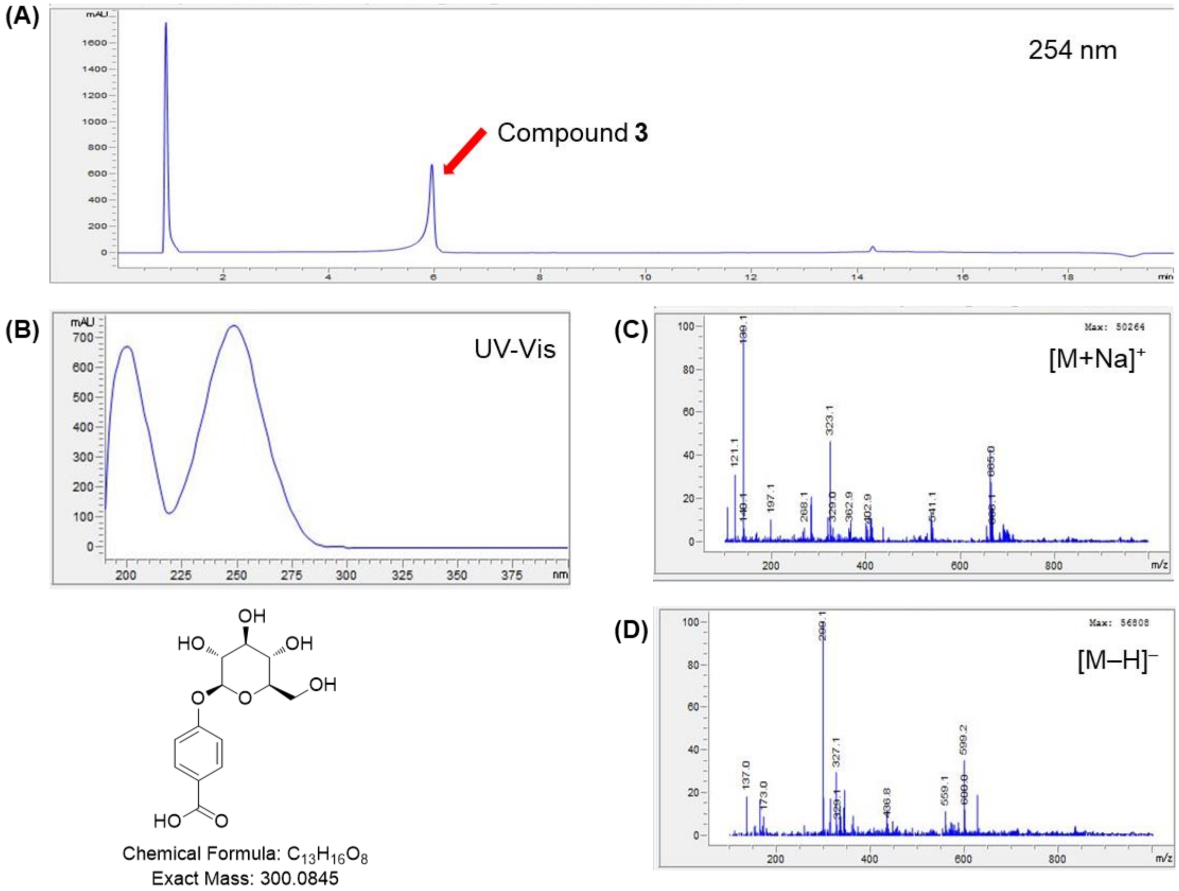

Figure S7. UV chromatogram of LC/MS (A: 254 nm) and UV (B) and MS data (C: positive; D: negative) for **3**.

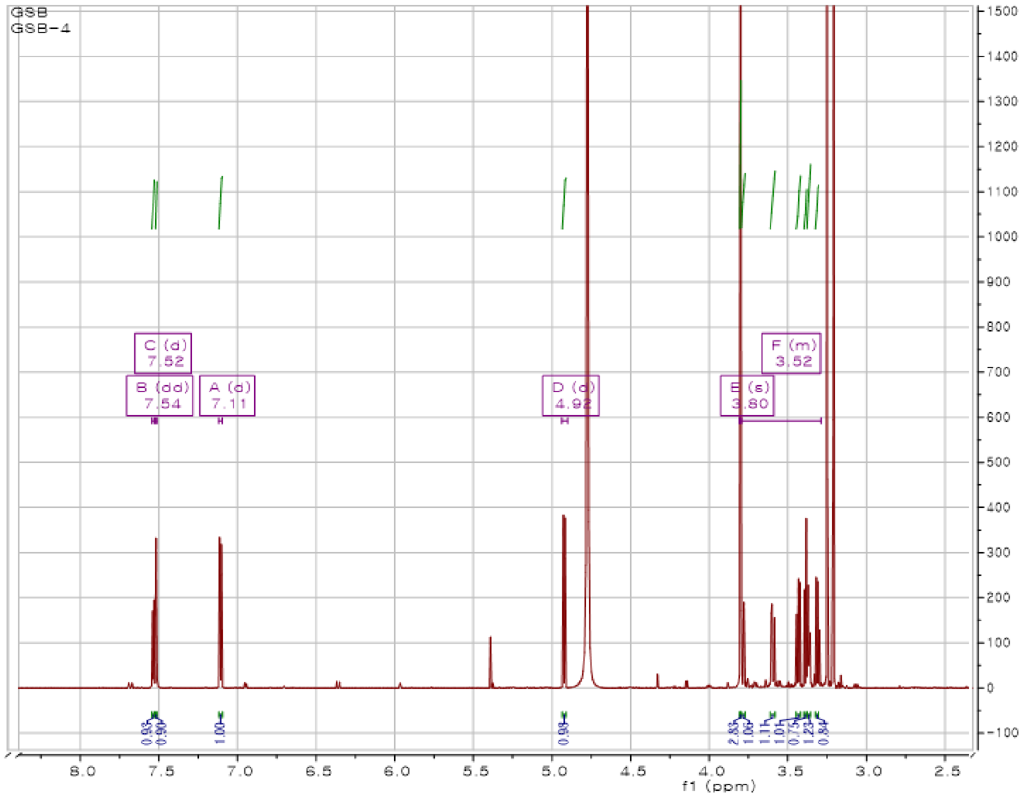

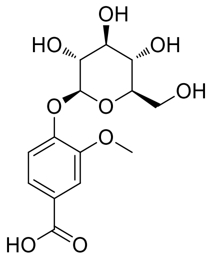

4

Figure S8. ^1H-NMR (CD_3OD, 850 MHz) spectrum of vanillic acid 4-*O*-β-D-glucopyranoside (**4**).

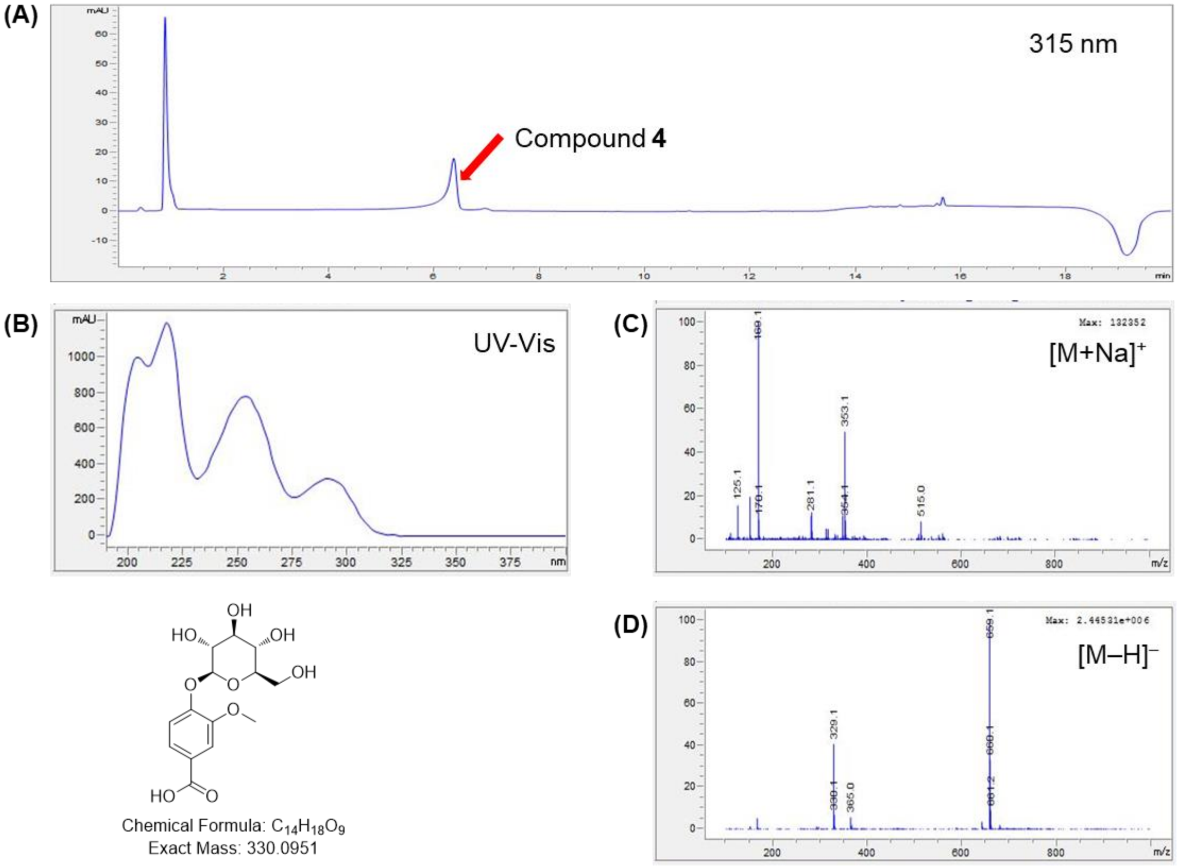

Figure S9. UV chromatogram of LC/MS (A: 315 nm) and UV (B) and MS data (C: positive; D: negative) for **4**.

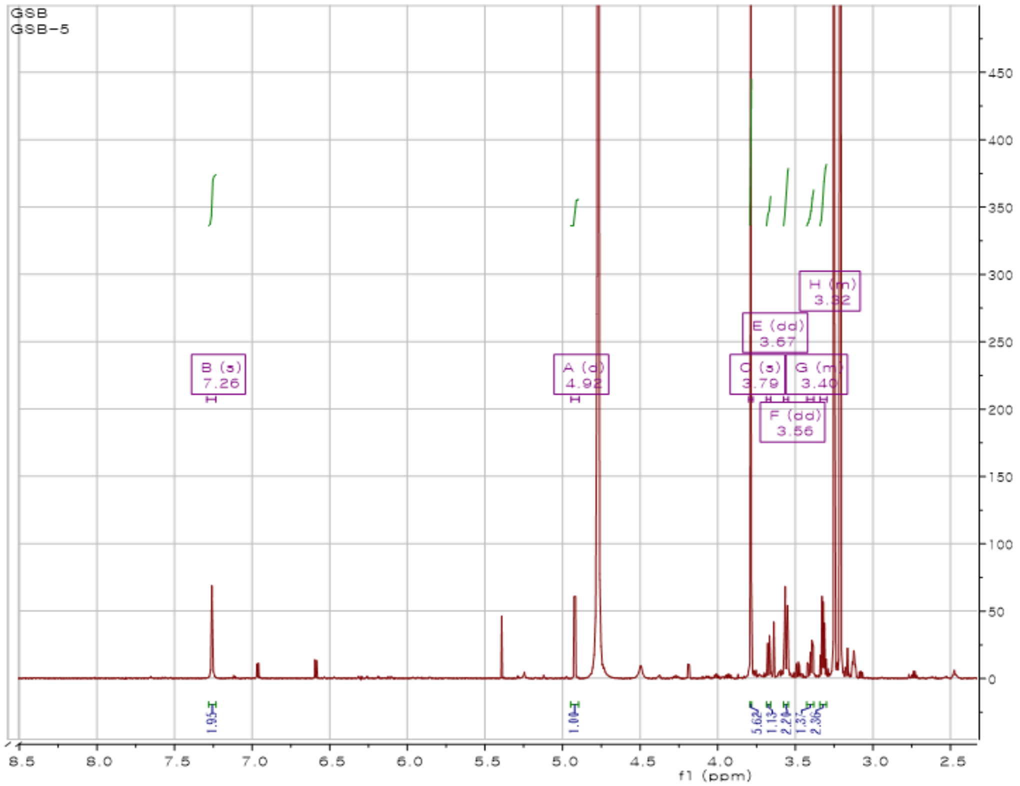

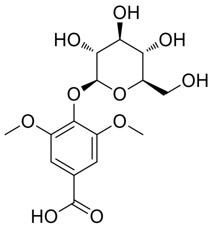

5

Figure S10. ^{1}H-NMR (CD_3OD, 850 MHz) spectrum of syringic acid 4-*O*-β-D-glucopyranoside (**5**).

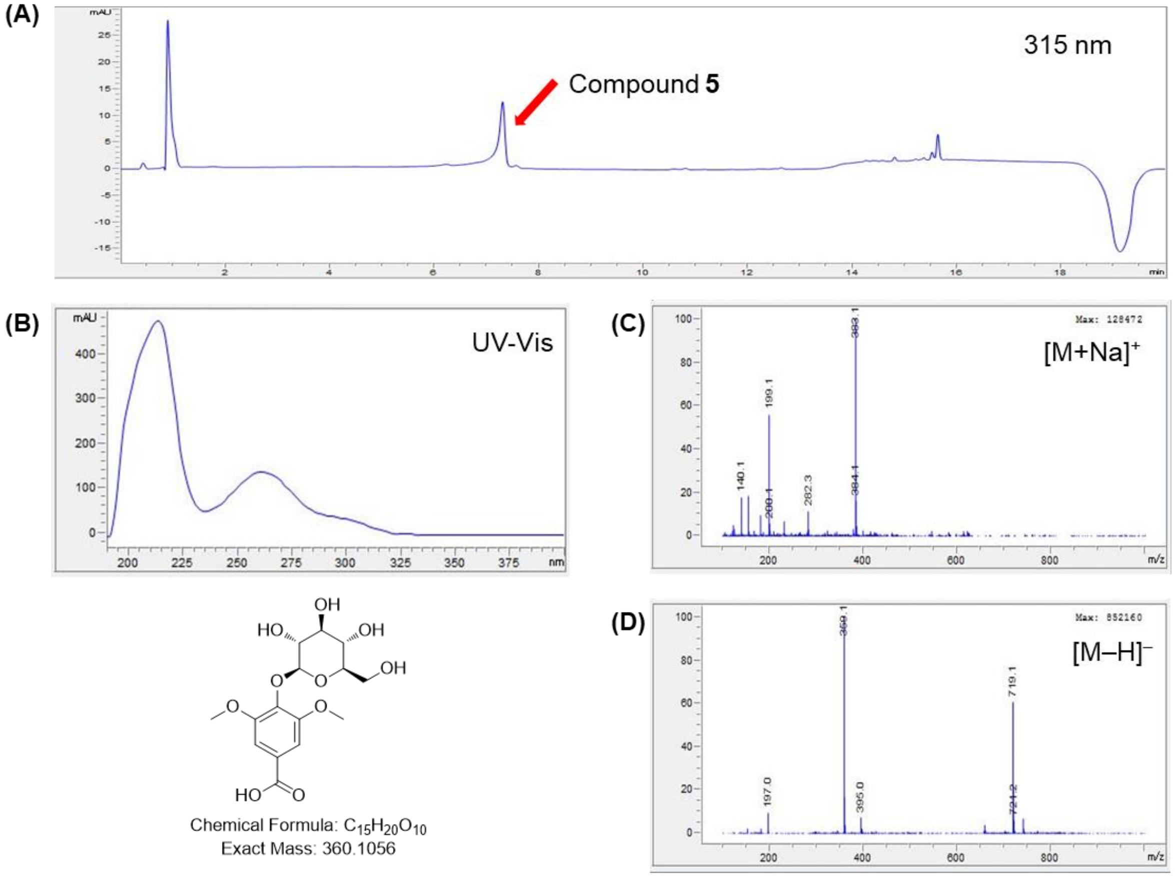

Figure S11. UV chromatogram of LC/MS (A: 315 nm) and UV (B) and MS data (C: positive; D: negative) for **5**.

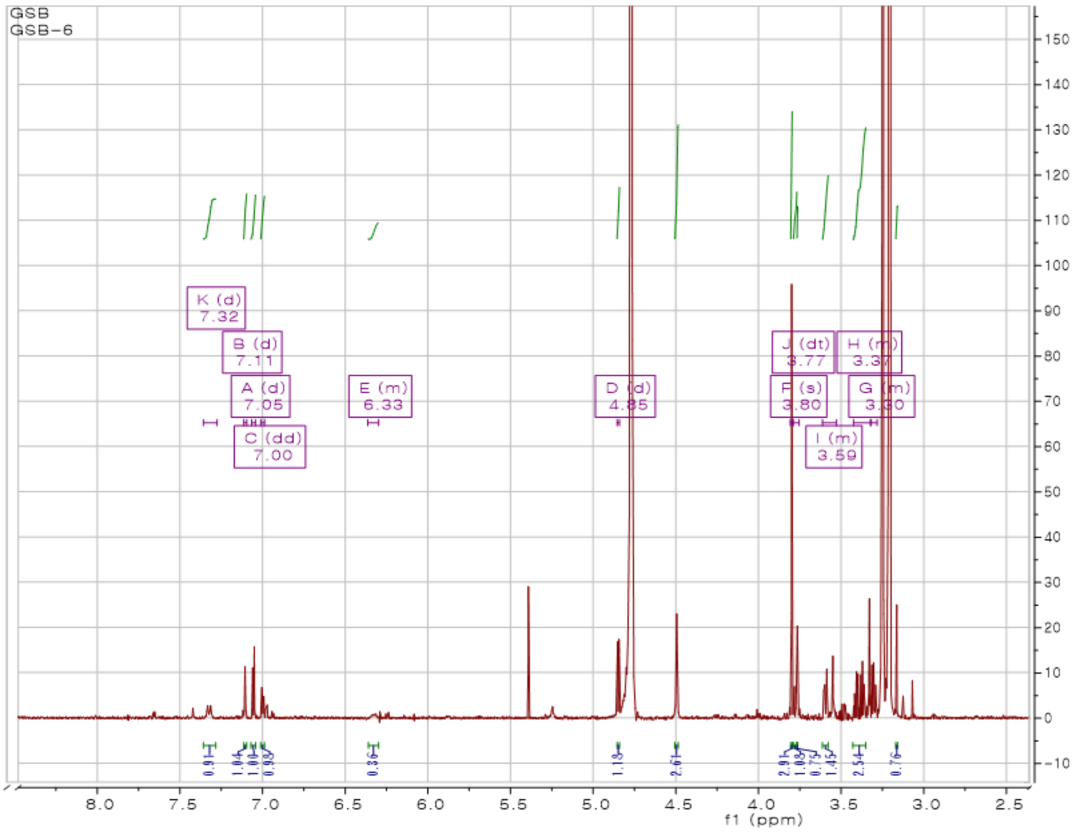

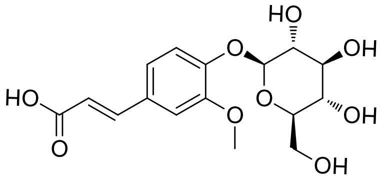

6

Figure S12. ^{1}H-NMR (CD_3OD, 850 MHz) spectrum of (*E*)-ferulic acid 4-*O*-β-D-glucoside (**6**).

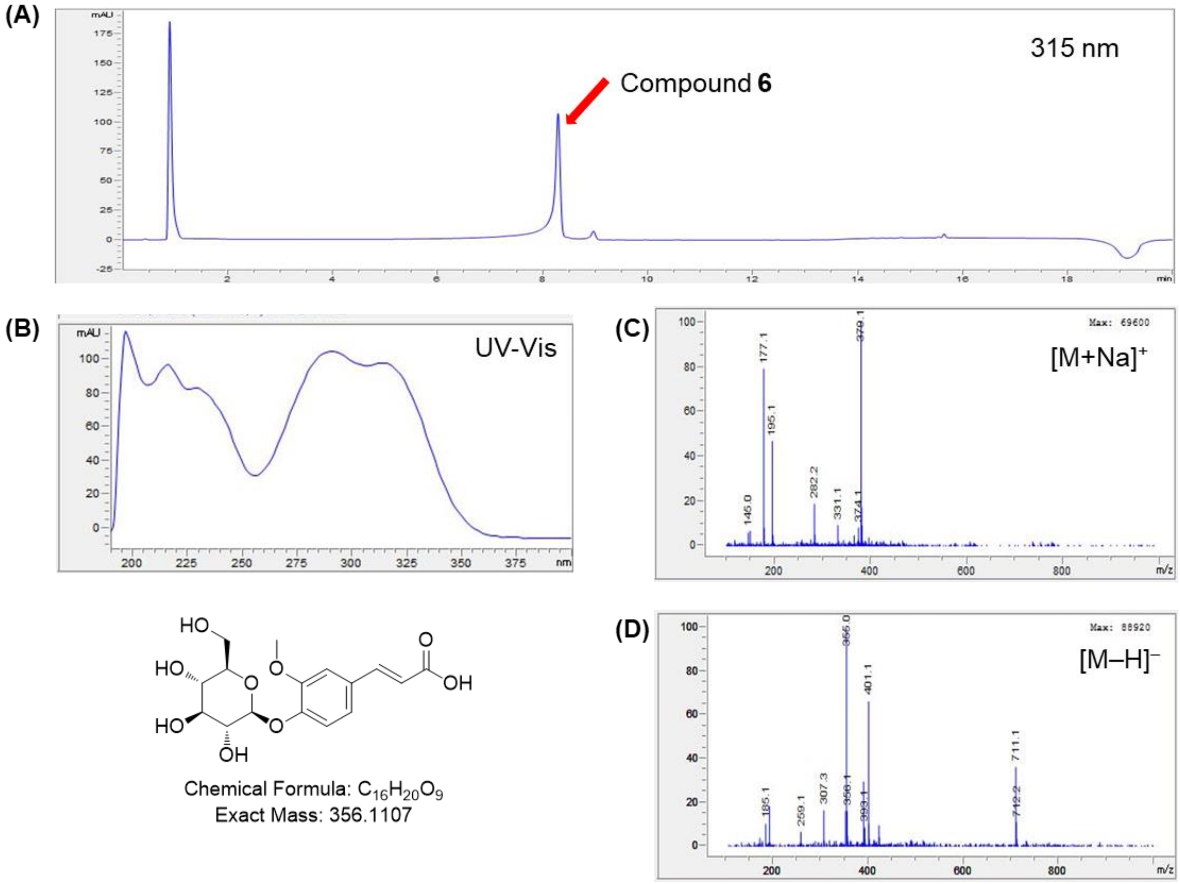

Figure S13. UV chromatogram of LC/MS (A: 315 nm) and UV (B) and MS data (C: positive; D: negative) for **6**.

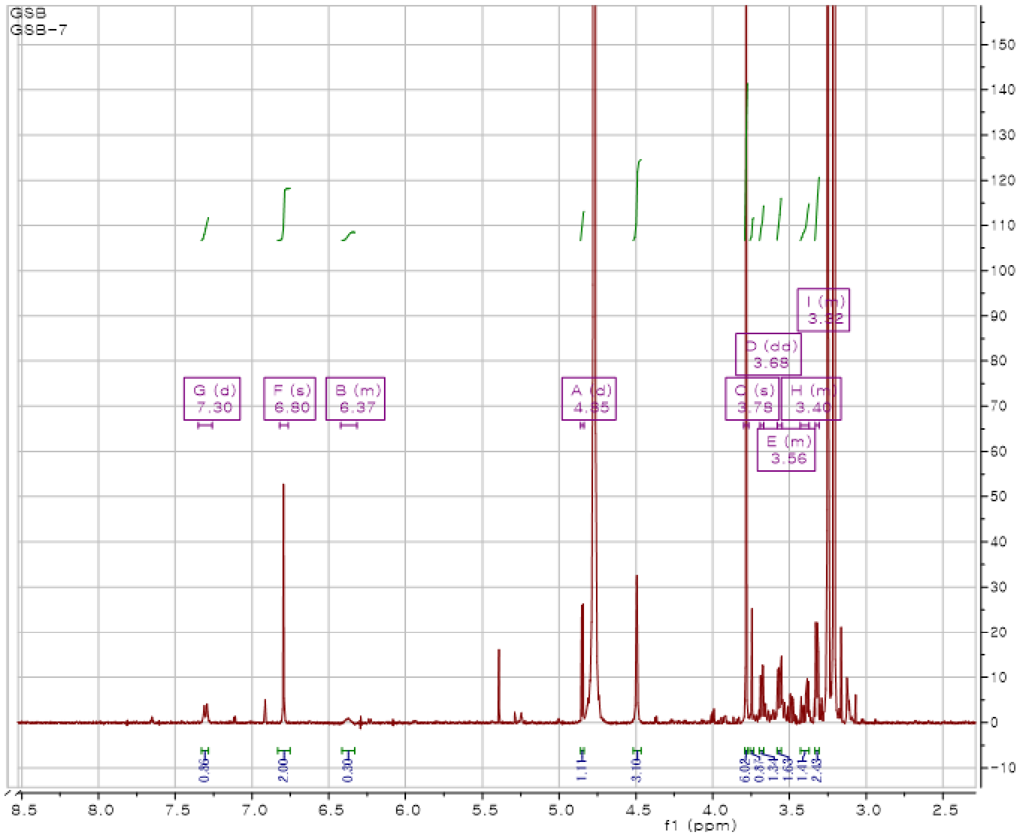

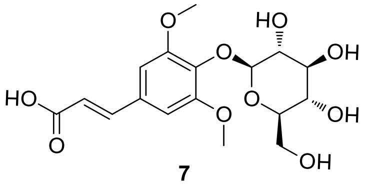

7

Figure S14. ^{1}H-NMR (CD_3OD, 850 MHz) spectrum of (*E*)-sinapic acid 4-*O*-β-D-glucopyranoside (**7**).

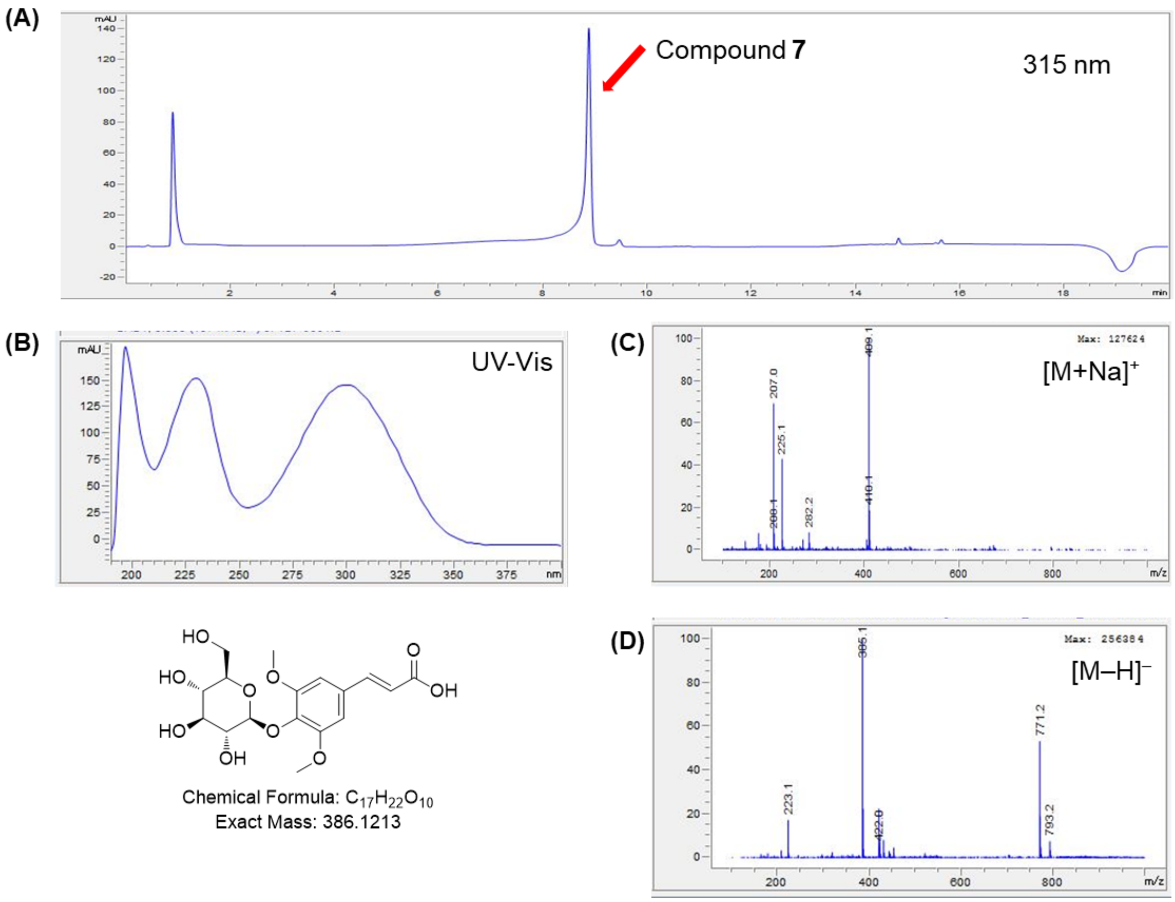

Figure S15. UV chromatogram of LC/MS (A: 315 nm) and UV (B) and MS data (C: positive; D: negative) for **7**.

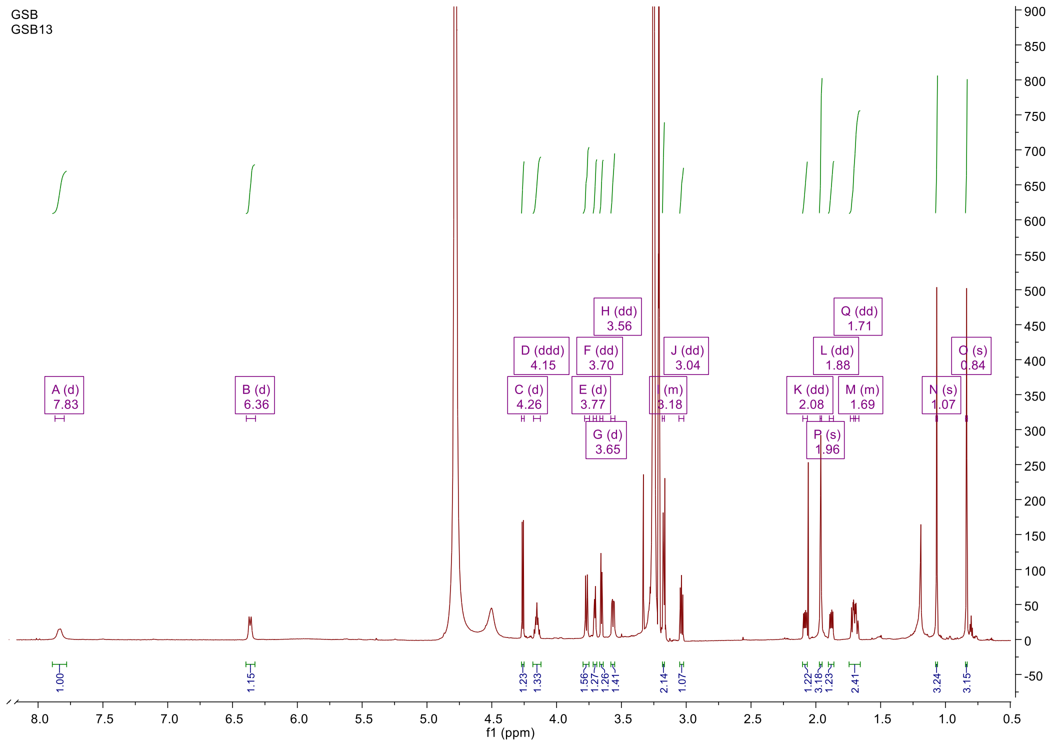

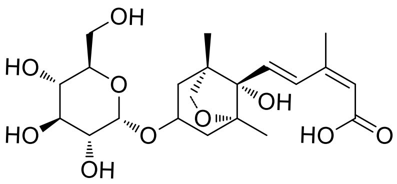

8

Figure S16. ^{1}H-NMR (CD_3OD, 850 MHz) spectrum of (1'*R*,2'*S*,5'*R*,8'*S*,2'*Z*,4'*E*)-dihydrophaseic acid 3'-*O*-β-D-glucopyranoside (**8**).

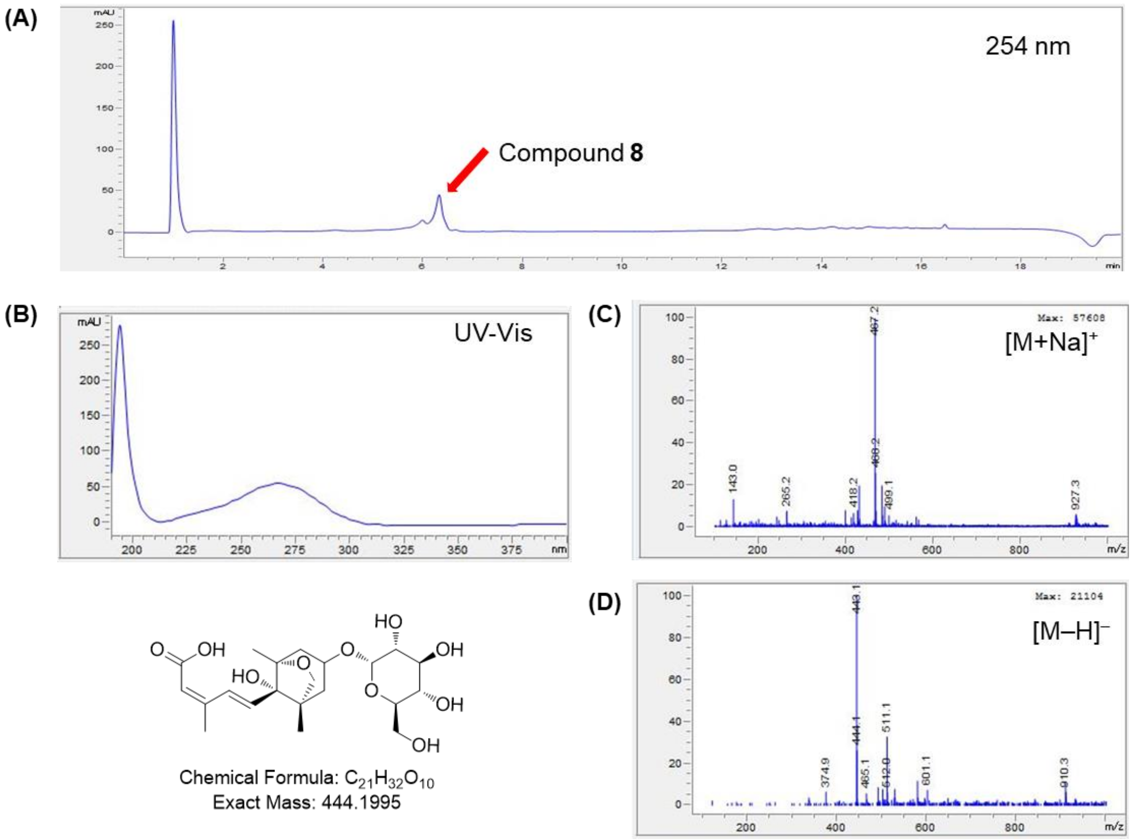

Figure S17. UV chromatogram of LC/MS (A: 254 nm) and UV (B) and MS data (C: positive; D: negative) for **8**.

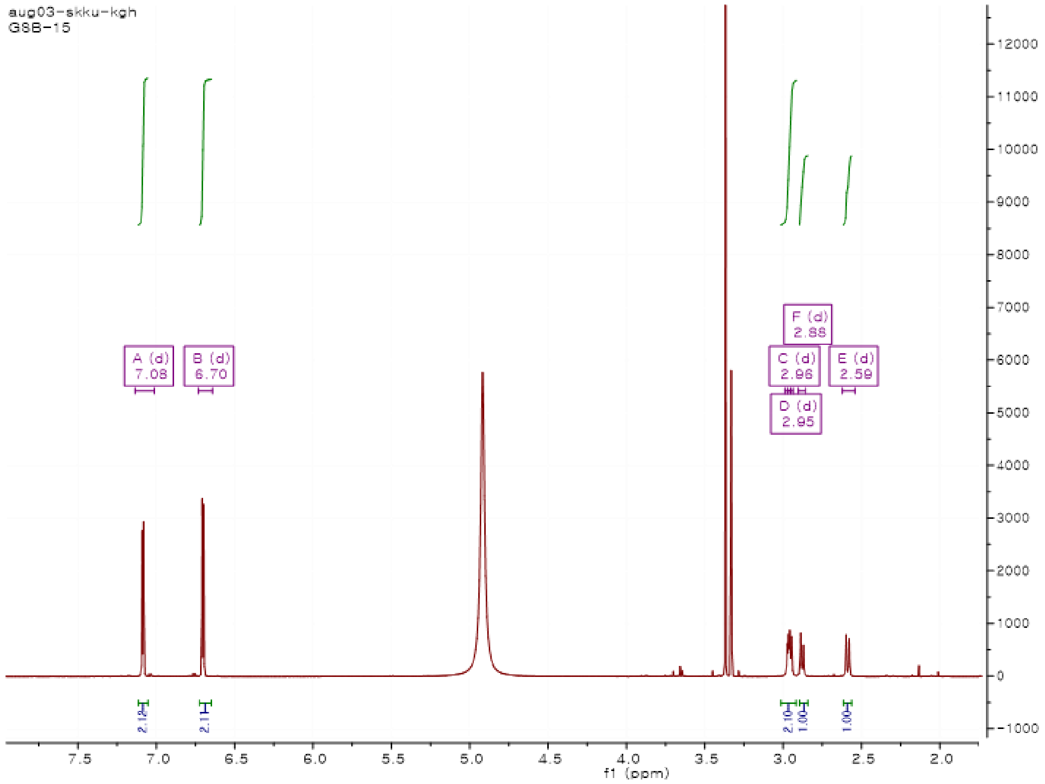

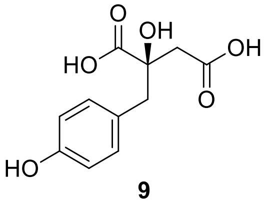

9

Figure S18. ^{1}H-NMR (CD_3OD, 850 MHz) spectrum of eucomic acid (**9**).

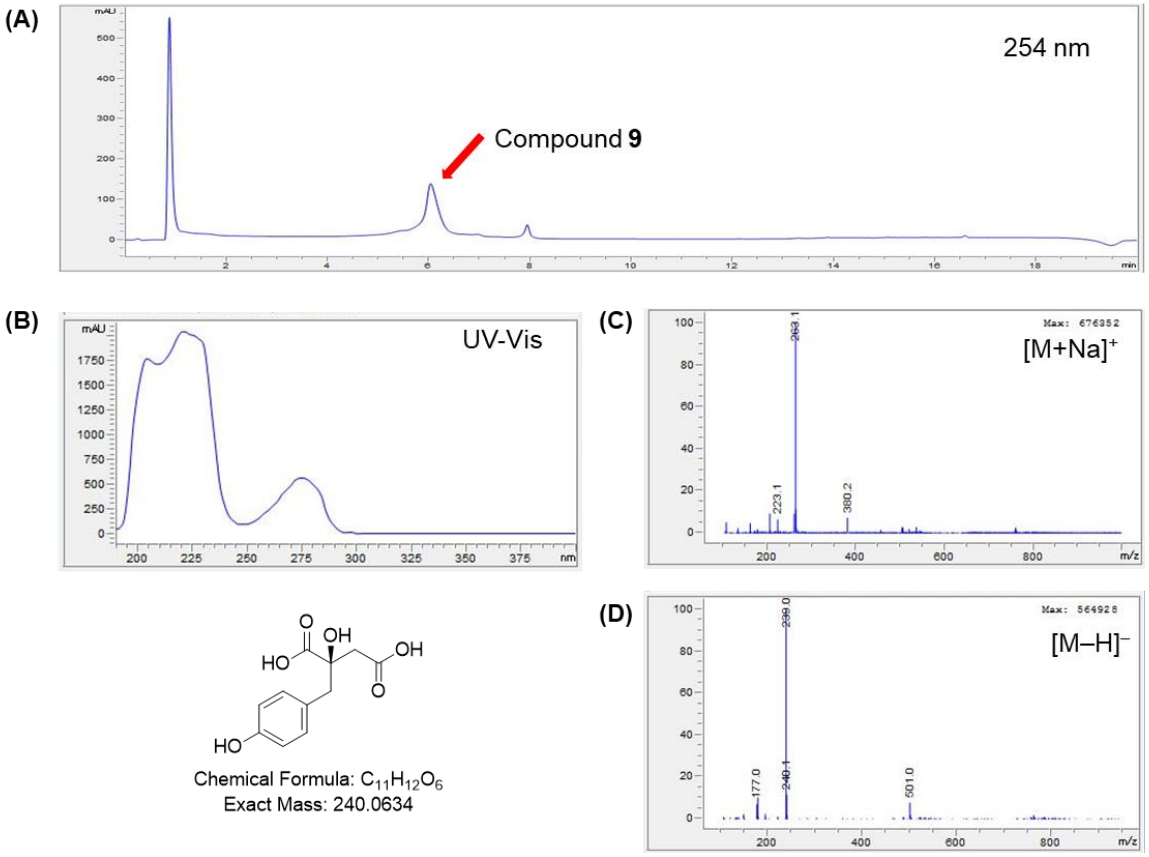

Figure S19. UV chromatogram of LC/MS (A: 254 nm) and UV (B) and MS data (C: positive; D: negative) for **9**.

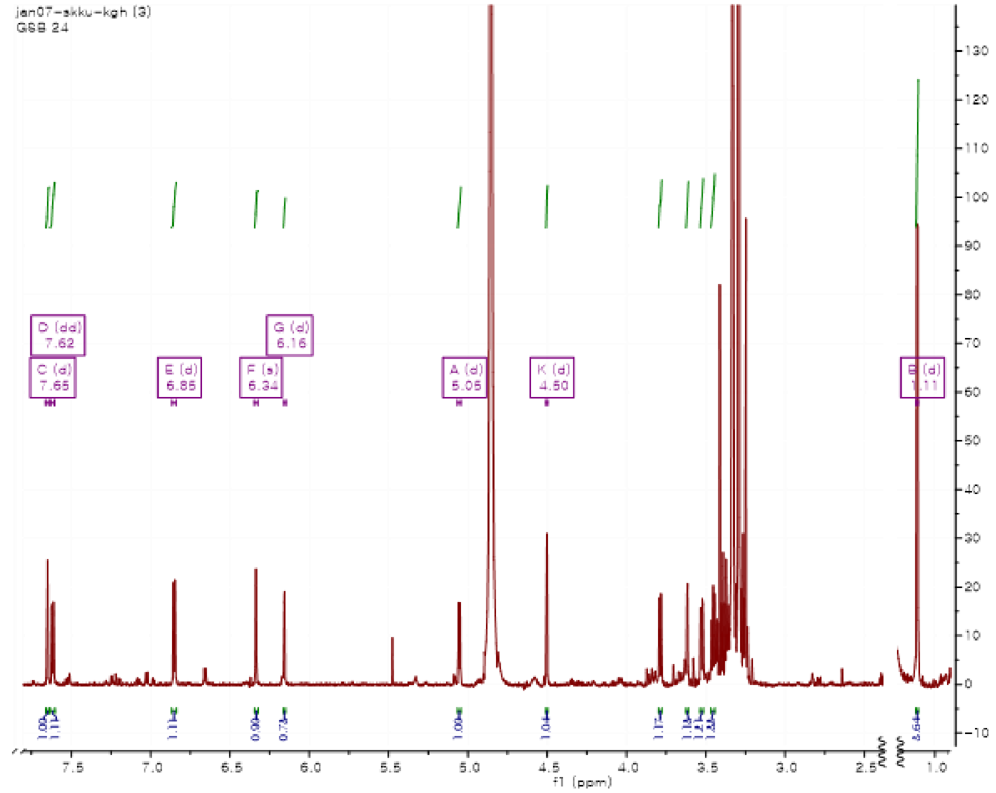

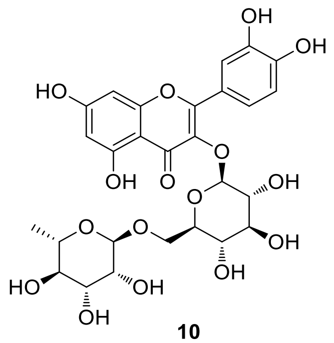

10

Figure S20. ^{1}H-NMR (CD_3OD, 850 MHz) spectrum of rutin (**10**).

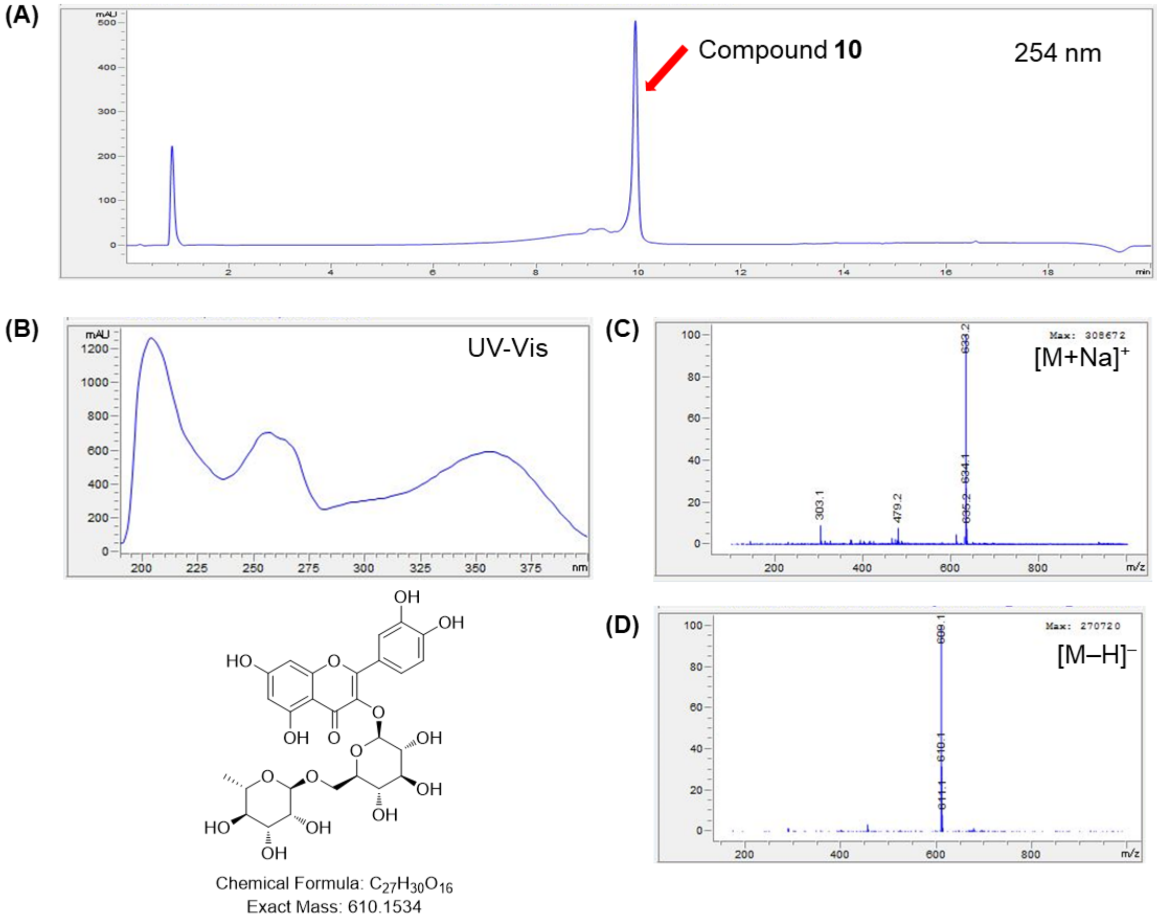

Figure S21. UV chromatogram of LC/MS (A: 254 nm) and UV (B) and MS data (C: positive; D: negative) for **10**.

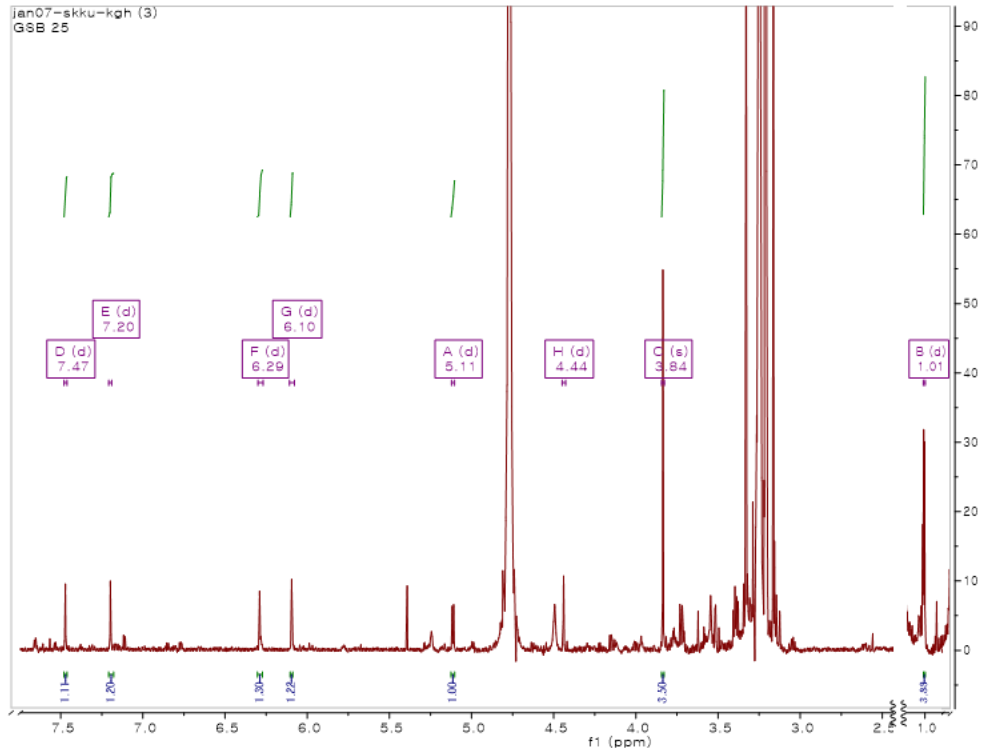

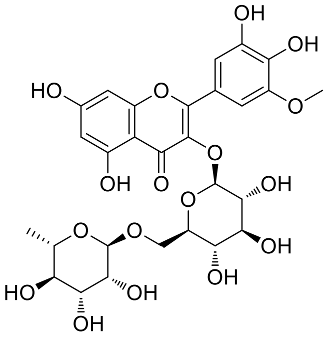

11

Figure S22. ^{1}H-NMR (CD_3OD, 850 MHz) spectrum of laricitrin 3-rutinoside (**11**).

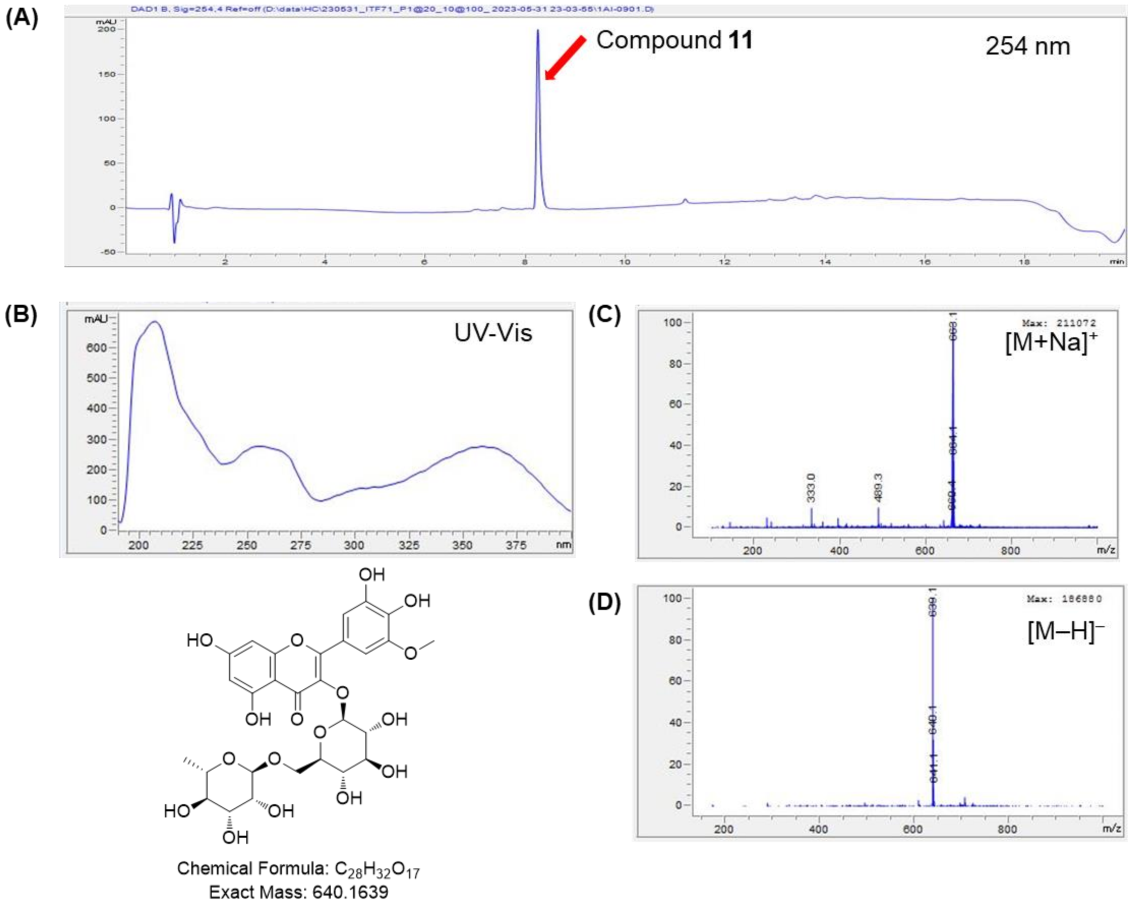

Figure S23. UV chromatogram of LC/MS (A: 254 nm) and UV (B) and MS data (C: positive; D: negative) for **11**.

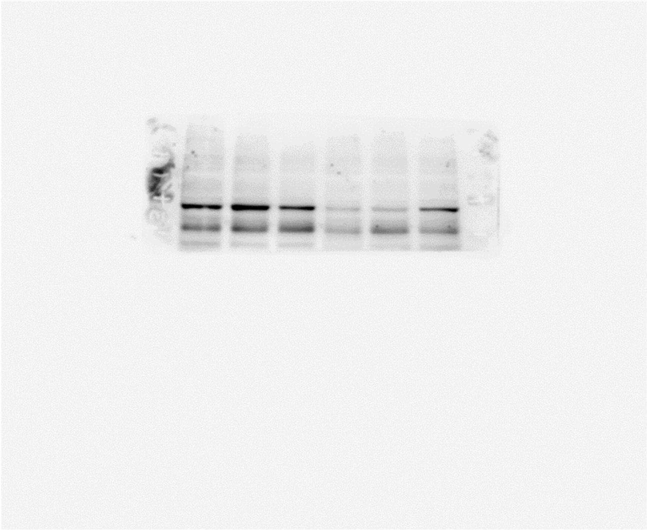

For ER-α protein

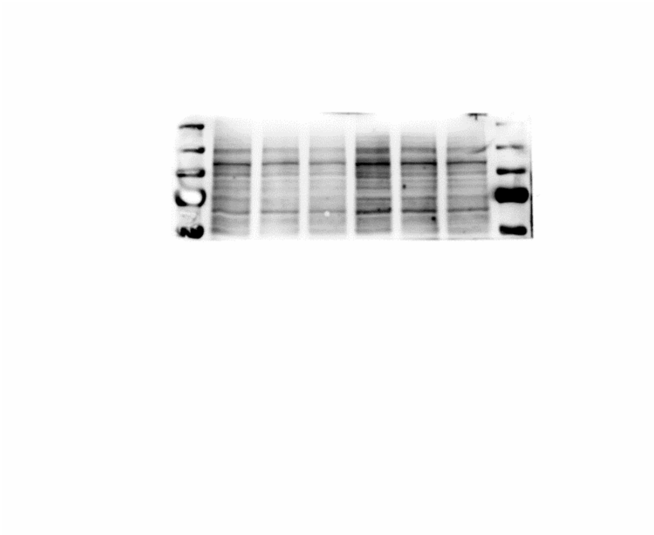

For Phospho-ER-α (Ser118) protein

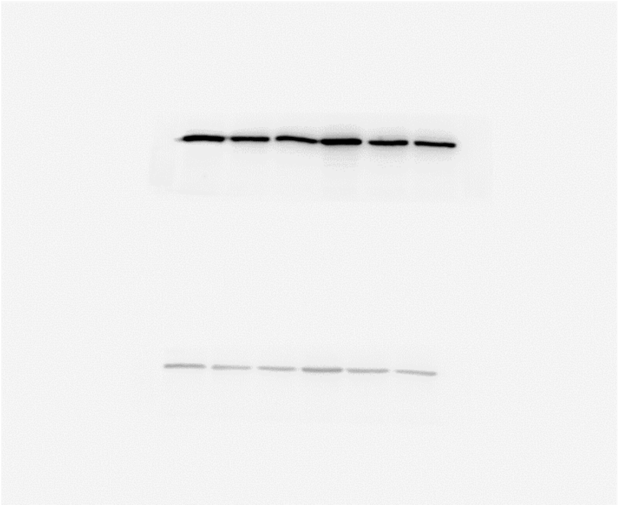

For GAPDH

Figure S24. The uncropped western blot gels

General Experimental Procedure

Optical rotations were measured using a Jasco P-2000 polarimeter (Jasco, Easton, MD, USA). Infrared (IR) spectra were recorded with a Bruker IFS-66/S FT-IR spectrometer (Bruker, Karlsruhe, Germany). Ultraviolet (UV) spectra were obtained using an Agilent 8453 UV-visible spectrophotometer (Agilent Technologies, Santa Clara, CA, USA). NMR spectra were acquired using a Bruker AVANCE III HD 850 NMR spectrometer, equipped with a 5 mm TCI CryoProbe operating at 850 MHz (^{1}H) and 212.5 MHz (^{13}C). Chemical shifts were reported in ppm (δ) for both ^{1}H and ^{13}C nuclear magnetic resonance (NMR) analyses. Preparative and semi-preparative high-pressure liquid chromatography (HPLC) were performed on a Waters 1525 Binary HPLC pump with a Waters 996 photodiode array detector (Waters Corporation, Milford, MA, USA). We employed an Agilent Eclipse C18 column (250 × 21.2 mm, 5 μm; flow rate: 5 mL/min; Agilent Technologies) and a Phenomenex Luna Phenyl-hexyl 100 Å column (250 × 10 mm, 5 μm; flow rate: 2 mL/min; Phenomenex, Torrance, CA, USA) for chromatography. Liquid chromatography-mass spectrometry (LC-MS) analyses were carried out using an Agilent 1200 Series HPLC system, equipped with a diode array detector, 6130 Series Electrospray Ionizing (ESI) mass spectrometer, and an analytical Kinetex C18 100 Å column (100 × 2.1 mm, 5 μm; flow rate: 0.3 mL/min; Phenomenex). High-resolution electrospray ionization mass spectrometry (HRESIMS) data were obtained on an Agilent G6545B quadrupole time-of-flight (Q-TOF) mass spectrometer (Agilent Technologies). Silica gel 60 (230–400 mesh; Merck, Darmstadt, Germany) was employed for column chromatography, while Diaion HP-20 (Mitsubishi Chemical, Tokyo, Japan) was used for open-column chromatography. Thin-layer chromatography was conducted on pre-coated silica gel F254 plates and RP-C18 F254s plates (Merck), with spot detection performed under UV light or by heating after spraying with anisaldehyde-sulfuric acid.
